# Aged‐vascular niche hinders osteogenesis of mesenchymal stem cells through paracrine repression of Wnt‐axis

**DOI:** 10.1111/acel.14139

**Published:** 2024-04-05

**Authors:** Viviane Fleischhacker, Filip Milosic, Marko Bricelj, Kristina Kührer, Katharina Wahl‐Figlash, Patrick Heimel, Andreas Diendorfer, Eleonora Nardini, Irmgard Fischer, Herbert Stangl, Peter Pietschmann, Matthias Hackl, Roland Foisner, Johannes Grillari, Markus Hengstschläger, Selma Osmanagic‐Myers

**Affiliations:** ^1^ Center for Pathobiochemistry and Genetics Medical University of Vienna Vienna Austria; ^2^ Department of Pathophysiology and Allergy Research, Center for Pathophysiology, Infectiology and Immunology Medical University of Vienna Vienna Austria; ^3^ Ludwig Boltzmann Institute for Traumatology (The Research Center in Cooperation with AUVA) Vienna Austria; ^4^ Austrian Cluster for Tissue Regeneration Vienna Austria; ^5^ Core Facility Hard Tissue and Biomaterial Research, Karl Donath Laboratory University Clinic of Dentistry, Medical University of Vienna Vienna Austria; ^6^ TAmiRNA GmbH Vienna Austria; ^7^ Max Perutz Labs, Vienna BioCenter Campus (VBC) Vienna Austria; ^8^ Max Perutz Labs Medical University of Vienna Vienna Austria; ^9^ Department of Biotechnology, Institute of Molecular Biotechnology University of Natural Resources and Life Sciences Vienna Austria

**Keywords:** aging, bone, cellular senescence, mesenchymal stem cells, microRNA, progeria, vascular

## Abstract

Age‐induced decline in osteogenic potential of bone marrow mesenchymal stem cells (BMSCs) potentiates osteoporosis and increases the risk for bone fractures. Despite epidemiology studies reporting concurrent development of vascular and bone diseases in the elderly, the underlying mechanisms for the vascular‐bone cross‐talk in aging are largely unknown. In this study, we show that accelerated endothelial aging deteriorates bone tissue through paracrine repression of Wnt‐driven‐axis in BMSCs. Here, we utilize physiologically aged mice in conjunction with our transgenic endothelial progeria mouse model (Hutchinson‐Gilford progeria syndrome; HGPS) that displays hallmarks of an aged bone marrow vascular niche. We find bone defects associated with diminished BMSC osteogenic differentiation that implicate the existence of angiocrine factors with long‐term inhibitory effects. microRNA‐transcriptomics of HGPS patient plasma combined with aged‐vascular niche analyses in progeria mice reveal abundant secretion of Wnt‐repressive microRNA‐31‐5p. Moreover, we show that inhibition of microRNA‐31‐5p as well as selective Wnt‐activator CHIR99021 boosts the osteogenic potential of BMSCs through de‐repression and activation of the Wnt‐signaling, respectively. Our results demonstrate that the vascular niche significantly contributes to osteogenesis defects in aging and pave the ground for microRNA‐based therapies of bone loss in elderly.

## Graphical abstract

Aging of the vascular niche hinders osteogenic differentiation of bone marrow mesenchymal stem cells (BMSCs) leading to a decline in osteogenesis and cortical bone thinning. Mechanistically, paracrine senescence‐associated microRNA‐31‐5p (miR‐31)‐signals derived from the aged‐vascular niche inhibit pro‐osteogenic Wnt signaling. Targeting microRNA‐31‐5p may represent a promising strategy to combat bone loss in elderly.

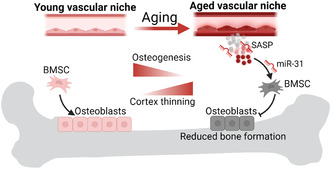

**Abbreviations**
Alpl: alkaline phosphatase
ARS: Alizarin Red S
BM: bone marrow
BMD: bone mineral density
bmECs: bone marrow endothelial cells
BMSCs: bone marrow mesenchymal stem cells
BV: bone volume
cb: cortical bone
CM: conditioned media
Col1a1: collagen type1 a1
Ct.Th: cortical thickness
Ctsk: cathepsin K
DDAOG: 9H‐(1,3‐dichloro‐9,9‐dimethylacridin‐2‐one‐7‐yl) β‐D‐galactoside
Dmp1: dentin‐matrix protein 1
dox: doxorubicin
ECs: endothelial cells
Emcn: endomucin
es: endosteum
Fzd3: frizzled class receptor 3
GSK3β: glycogen synthase 3β
HE: hematoxylin and eosin
HGPS: Hutchinson‐Gilford progeria syndrome
hLMNA: anti‐human lamin A antibody
HSCs: hematopoietic stem cells
LA‐Tg: endothelial wild‐type human lamin A mouse model
Lef1: lymphoid enhancer factor 1
Lepr: leptin receptor
LM_LA‐Tg: littermate LA‐Tg controls
Mepe: matrix extracellular phosphoglycoprotein
miR: microRNA
MP: metaphysis
Nfatc1: nuclear factor of activated T cells
PINP: procollagen type I N‐terminal propeptide
Prog‐Tg: endothelial progeria mouse model
ps: periosteum
Runx2: runt‐related transcription factor 2
SA‐miR: senescence‐associated microRNAs
SASP: senescence‐associated secretory phenotype
SA‐β‐gal: senescence‐associated beta‐galactosidase
TRAP: tartrate‐resistant acid phosphatase
TV: total volume
Wt: wild‐type
μCT: micro‐computed tomography

## 1 INTRODUCTION

Aging is a gradual process that leads to a progressive decline in tissue function associated with the development of age‐related diseases. The skeleton is especially affected in the elderly population associated with osteoporosis development, increased bone fracture risk, mobility loss, and thus impaired quality of life (Doolittle et al., 2021). Bone is a highly vascularized tissue, composed of a broad vascular network of large vessels and small capillaries (Kusumbe et al., 2014). Changes in the vasculature in atherosclerosis have been linked with osteoporosis in epidemiology studies (Mo et al., 2022), however, the molecular mechanisms underlying vascular‐dependent osteoporotic changes during aging are mostly unexplored. In this regard, angiocrine factors may play an important role enabling a tight interaction between endothelial cells (ECs) of the bone marrow (BM) and bone tissue (Zhu et al., 2020). The regulatory role of such angiocrine factors, coupling angiogenesis with osteogenesis, has been reported in hematopoiesis but their mode of action and paracrine effects on bone marrow mesenchymal stem cells (BMSCs) are mostly unknown (Biswas et al., 2023; Kusumbe et al., 2014).

During aging certain stem cell subpopulations exhibit a decline in regenerative potential due to reduced self‐renewal and differentiation capacity ultimately leading to tissue deterioration (Brunet et al., 2022). Most of the work so far focused on functional changes in hematopoietic stem cells (HSCs) showing that HSCs undergo clonal expansion associated with the production of fewer lymphoid progenitors causing myeloid skewing (Brunet et al., 2022; Ho et al., 2019). As for BMSCs, aging was primarily associated with reduced number and osteogenic differentiation capacity and a bias toward adipogenic lineage promoting osteoporosis development (Wang et al., 2022).

Stem cells are maintained and regulated in specialized microenvironments, so‐called niches that secrete extrinsic factors to maintain stem cell homeostasis (Brunet et al., 2022). In recent years the bone marrow “vascular niche” has gained interest in the context of HSCs with an emerging role for bone lineage cells as well (Chen et al., 2020; He et al., 2014). BM vascular niche comprises heterogenous bone vasculature such as bone vessels lining endosteal surfaces, sinusoidal vasculature in the bone marrow cavity, and presumably type H vessels for bone lineage cells. Aging is associated with changes in secreted extrinsic factor patterns from the niche cells and altered cell–cell communication with stem cells (Brunet et al., 2022). However, how aging alters signals of the BM vascular niche released into the microenvironment and the impact of these messages on BMSCs studied in in vivo context is largely missing.

To study the effects of an aged‐vascular niche on BMSCs and bone structure we utilized our previously generated endothelial progeria mouse model (*Prog‐Tg*) that displays hallmarks of premature aging in the endothelial tissue (Manakanatas et al., 2022; Osmanagic‐Myers et al., 2019). *Prog‐Tg* mice express selectively in the endothelial tissue, progerin, which is a mutated form of the lamin A protein (c.1824 C>T, pG608G) known to cause a devastating premature aging disease Hutchinson‐Gilford progeria syndrome (HGPS) (Eriksson et al., 2003). HGPS patients and mouse models develop typical premature aged features and age‐related disorders such as atherosclerosis, cardiovascular disease, and osteoporosis associated with low bone mass, increased fracture risk, and osteolysis of the ribs (Hamczyk et al., 2018; Merideth et al., 2008; Osmanagic‐Myers et al., 2019). On the cellular level, progerin‐expressing cells display typical aging hallmarks (Lopez‐Otin et al., 2023) such as epigenetic changes, reduction in telomere length, increased DNA damage, and genomic instability associated with the development of stable cell cycle arrest named cellular senescence (Cenni et al., 2020; Manakanatas et al., 2022; Mojiri et al., 2021; Primmer et al., 2022). Accordingly, we and others could demonstrate that progerin expression in ECs of *Prog‐Tg* mice results in the accumulation of senescent ECs in tissues with similar findings in human ECs derived from induced pluripotent stem cells of HGPS children (Manakanatas et al., 2022; Mojiri et al., 2021). This is accompanied by the development of senescence‐associated secretory phenotype (SASP) including a release of senescence‐associated microRNAs (SA‐miR) into the circulation with potentially systemic tissue‐damaging effects (Manakanatas et al., 2022).

Here, we show for the first time that *Prog‐Tg* mice display characteristics of pathologically aged BM vascular niche with long‐term deteriorating effects on the osteogenic differentiation capacity of BMSCs. A significant increase in inflammation and senescence of the BM microenvironment is demonstrated in line with bone loss, and cortical thinning. Mechanistically, our data highlight cell non‐autonomous repressive effects of endothelial‐derived circulatory SA‐miR‐31 on Wnt‐mediated Lef1 signaling hindering BMSC differentiation to osteogenic lineage.

## 2 RESULTS

### 2.1 Endothelial progeria mice exhibit altered bone microstructure

**FIGURE 1:**
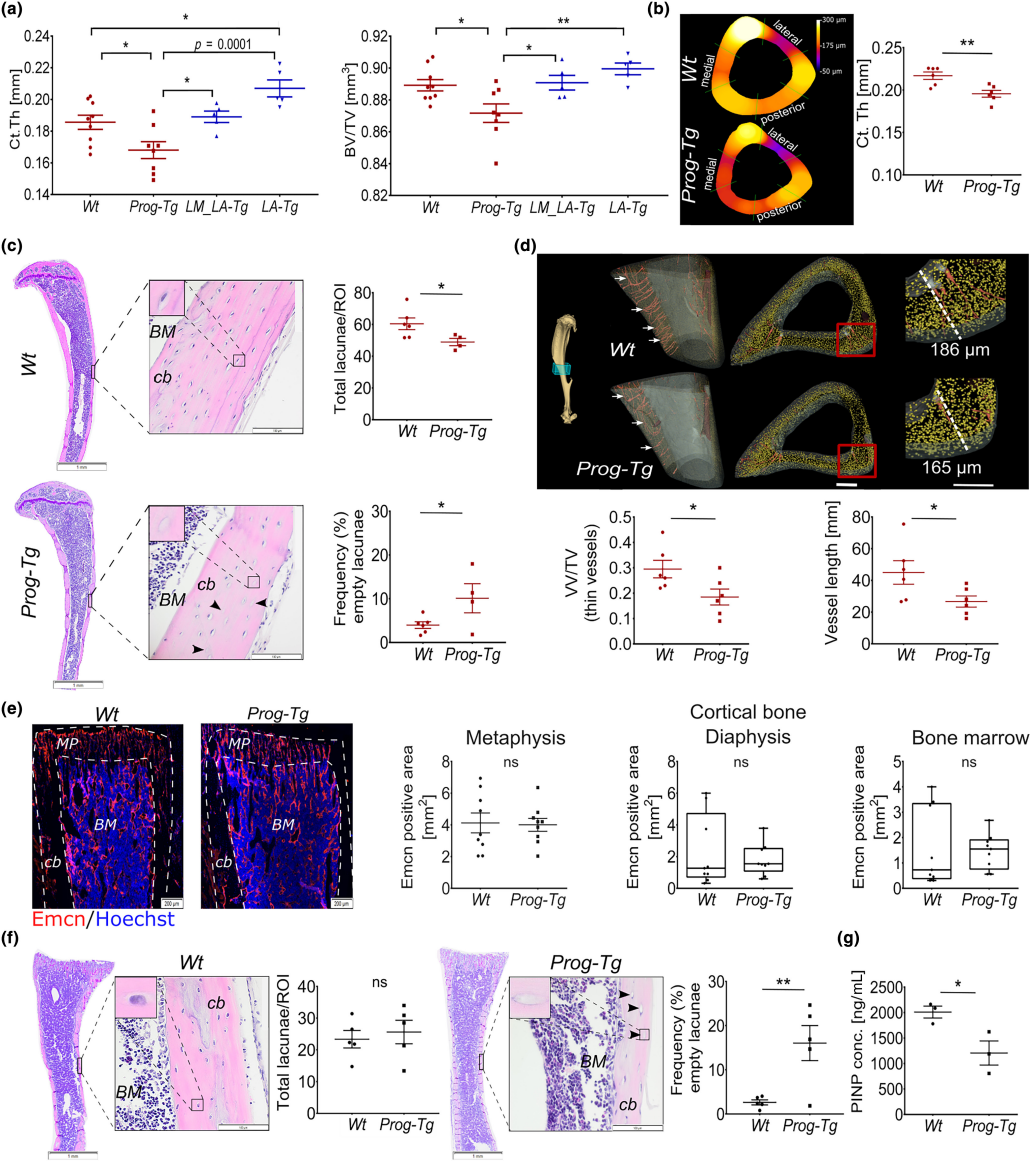
Cortical bone thinning in *Prog‐Tg* mice. (a) Quantification of average cortical thickness (Ct.Th) in mm, and bone volume (BV) to total volume (TV) ratio using micro‐computed tomography of tibiae in *Prog‐Tg* (*n* ≥ 8), *LA‐Tg* (*n* = 5) and corresponding single‐transgenic littermates, *Wt* and *LM_LA‐Tg*, respectively (age = 35–40 weeks). One‐way ANOVA with post hoc Tukey's multiple comparisons test. (b) Representative cross‐sections of nanoCT images of tibiae with quantification of average Ct.Th in *Wt* and *Prog‐Tg* animals (*n* = 6; age = 30–35 weeks). (c) Quantification of total osteocyte lacunae as mean number per region of interest (ROI) including empty osteocyte lacunae (arrows) shown as proportion of total in [%] on HE‐stained cortical tibiae sections of *Wt* and *Prog‐Tg* (age = 30–35 weeks). Each data point represents mean values from one animal (*n* = 4–6). BM, bone marrow; cb, cortical bone. Scale bars, 1 mm and 100 μm. (d) Representative reconstructed 3D nanoCT images of tibiae regions marked within blue cube showing on the left panels reduced vascularization (arrows) and the right cortical thinning in *Prog‐Tg* compared to *Wt* animals (age = 30–35 weeks). Mean vascular volume fraction (VV/TV) and the mean length of blood vessels are shown below. Scale bar, 100 μm. (e) Representative images of endomucin (Emcn) stained vasculature in tibia sections from young *Wt* and *Prog‐Tg* animals (age ≤ 14 days, *n* = 9, with 3 biological replicates in 3 serial sections per animal). Mean endomucin positive area [%] is shown for metaphysis (MP), cortical bone (cb), and bone marrow (BM). (f) Total and empty osteocyte lacunae (see (c)) in cortical tibiae regions from young animals (age ≤ 14 days, *n* = 4–6). Scale bars, 1 mm and 100 μm. (g) Procollagen type I N‐terminal propeptide (PINP; ng/mL) protein levels in plasma from young animals analyzed by ELISA (age ≤ 14 days, *n* = 3). Unpaired two‐tailed Student's *t*‐test. (*p < 0.05, **p < 0.01, ns, not significant).

To determine if age‐related changes of the vascular endothelium affect bone tissue, we utilized our progeria mouse model (*Prog‐Tg*) displaying hallmarks of selective premature aging in the endothelial tissue (Manakanatas et al., 2022; Osmanagic‐Myers et al., 2019). *Prog‐Tg* mice were generated by crossing transgenic mice carrying tet‐op driven expression of mutated human lamin A minigene (1824C>T; p.G608G) (Sagelius et al., 2008) with endothelial transactivator mice (*Cdh5*‐tTA) (Sun et al., 2005). As controls for *Prog‐Tg* animals their corresponding single‐transgenic littermate animals were used that were referred as wild‐type (*Wt*) in the text (Figure S1). *LA‐Tg* mice were utilized as additional controls that showed transgenic expression of only wild‐type human lamin A minigene in endothelial tissue together with their corresponding single‐transgenic littermate controls referred to as *LM_LA‐Tg* (Figure S1; Osmanagic‐Myers et al., 2019). Micro‐computed tomography (μCT) of tibiae from *Prog‐Tg* mice revealed a significant reduction in average cortical bone thickness (Ct.Th) of ~14% and ~11% compared to *LA‐Tg* and *Wt* controls, respectively (Figure 1a and Table 1). This is consistent with the extent of mean Ct.Th decline observed in physiologically aged mice (0.17 mm at 22 weeks to 0.15 mm at 104 weeks) and ubiquitous prematurely aged HGPS mice as well as elderly patients (Cabral et al., 2023; Ferguson et al., 2003; Whitmarsh et al., 2019). Accordingly, bone volume fraction (BV/TV) is significantly reduced if compared to all three control groups indicating a profound effect of endothelial aging on bone (Figure 1a and Table 1). No reduction in BV/TV with even an increase in Ct.Th in *LA‐Tg* animals confirmed the specific effects of progerin (Figure 1a and Table 1). Furthermore, since previous findings have shown reduced body weights for *Prog‐Tg* animals (Osmanagic‐Myers et al., 2019), we measured the length of tibiae using μCT which have revealed similar lengths of tibiae in all four assessed groups (Table 1). Trabecular parameters were not altered in tibiae and fourth vertebral body of the lumbar spine indicating that selective endothelial aging does not lead to changes in the trabecular bone microstructure (Table S1). Since *LA‐Tg* control animals displayed no bone phenotype, we included them subsequently only in key experiments as additional controls in comparison to their respective *LM_LA‐Tg* littermates. Altogether, *Prog‐Tg* phenotype resembles cortical bone thinning observed in aged mice as well as elderly.

**TABLE 1:** Cortical bone parameters assessed by μCT measurements in tibia.

Tibia							
	*Wt*	*Prog‐Tg*	*p*‐Value *Wt* vs. *Prog‐Tg*	*LM_LA‐Tg*	*LA‐Tg*	*p*‐Value *LM_LA‐Tg* vs. *LA‐Tg*	*p*‐Value *Prog‐Tg* vs. *LA‐Tg*
TV [mm^3^]	0.53 ± 0.05	0.49 ± 0.07	ns	0.69 ± 0.06	0.66 ± 0.09	ns	ns
BV [mm^3^]	0.48 ± 0.05	0.43 ± 0.07	ns	0.61 ± 0.03	0.59 ± 0.08	ns	ns
BV/TV	0.89 ± 0.01	0.87 ± 0.02*	0.019*	0.89 ± 0.01	0.89 ± 0.01	ns	0.0033**
Ct.Th [μm]	186 ± 13	168 ± 14*	0.023*	189 ± 7	195 ± 20*	0.024*	0.001***
BMD [mgHA/ccm]	977 ± 21	957 ± 20	ns	968 ± 20	963 ± 22	ns	ns
Length [cm]	1.64 ± 0.13	1.54 ± 0.18	ns	1.57 ± 0.15	1.53 ± 0.15	ns	ns

*Note*: *Prog‐Tg*, *LA‐Tg*, and corresponding *Wt* littermates (*LM*) analyzed using μCt (age = 35–40 weeks; *n* = 8 *Prog‐Tg*, *n* = 9 *Wt* littermates (*Wt*), *n* = 5 *LA‐Tg*, and n = 5 for corresponding *Wt* littermate (*LM_LA‐Tg*)). Data presented as mean ± SD. Statistical analysis by one‐way ANOVA followed by multiple comparisons post hoc Tukey test (*p < 0.05, **p < 0.01, ***p < 0.001, ns, not significant).

Abbreviations: BMD, bone mineral density; BV, bone volume; BV/TV, cortical bone volume fraction; Ct.Th, average cortical thickness; TV, total volume.

Consistent with μCT data, nanoCT measurements for in‐depth analysis of bone nano‐architecture, confirmed a clear reduction of ~11% in average cortical thickness of *Prog‐Tg* animals (Figure 1b). Generally, we noticed a slight variation in cortical thickness values between μCT and nanoCT measurements that may have been caused by minor variation in the scanned tibia regions and different resolution of the techniques. To test if bone loss was more pronounced at particular regions, as previously described for ubiquitously aged mice (Ferguson et al., 2003), we measured average Ct.Th at bone regions that were defined based on the angle relative to the center of the medullary. Using a 60° split‐angle to define posterior, lateral, and medial bone regions, we found almost ~19% decline in Ct.Th at medial regions confirming preferential bone loss at these sites (Figure 1b, Table 2). Importantly, both methods confirmed reduced Ct.Th in *Prog‐Tg* animals strengthening the validity of our findings. Furthermore, hematoxylin and eosin (HE) staining of whole bone sections revealed significantly reduced numbers of osteocyte lacunae in cortices of *Prog‐Tg* mice with increased proportions of empty osteocyte lacunae indicating alterations in the cortical bone microstructure (Figure 1c). Thereby, we found no gross changes in the BM adiposity that would point to altered BMSC adipogenesis as an underlying cause for such changes (Figure S2a). Importantly, analysis of reconstructed 3D nanoCT images revealed significantly reduced mean vascular volume fraction (VV/TV) and reduced mean length of blood vessels suggesting that alterations of the vasculature cause bone defects in *Prog‐Tg* animals (Figure 1d).

**TABLE 2:** Cortical thickness in different regions of the tibia assessed by nanoCT measurements.

Tibia			
	*Wt*	*Prog‐Tg*	*p*‐Value *Wt* vs. *Prog‐Tg*
Lateral	0.1533 ± 0.0100	0.1345 ± 0.0092	0.0069**
Medial	0.1838 ± 0.0178	0.1549 ± 0.0139	0.0106*
Posterior	0.1835 ± 0.0240	0.1803 ± 0.0158	ns

*Note*: *Prog‐Tg* and corresponding *Wt* littermates analyzed using nanoCT (age = 30–35 weeks; *n* = 6 *Prog‐Tg*, *n* = 6 *Wt* littermates (*Wt*)). Data presented as mean ± SD. Statistical analysis by unpaired two‐tailed Student's *t*‐test (*p < 0.05, **p < 0.01, ns, not significant).

To address if changes in the bone microstructure may be secondary due to reduction in vascularization, we histologically assessed tibiae of young animals (age = 10–14 days) for changes in the bone microvessel density and bone phenotype. We found no changes in the density of endomucin (Emcn) stained bone microvessels within metaphysis, cortical regions of diaphysis, and BM while a significantly increased proportion of empty but not total osteocyte lacunae still indicated bone defects in young *Prog‐Tg* animals (Figure 1e,f). Empty osteocyte lacunae appeared not to be the consequence of increased osteocyte apoptosis since TUNEL staining on whole bone sections in young as well as adult animals was negative (Figure S2b). Significantly reduced levels of bone formation and remodeling marker procollagen type 1 N‐terminal propeptide, PINP1 (Weigl et al., 2021) rather points toward reduced bone formation and remodeling as a likely cause for empty osteocyte lacunae and bone defects in young *Prog‐Tg* animals (Figure 1g). Altogether, we concluded that changes in the bone microstructure are not entirely caused by vascular rarefication but rather pointed toward paracrine factors of the vascular niche mediating the effects on the bone.

### 2.2 Specific progerin expression causes senescence of the bone vasculature

**FIGURE 2:**
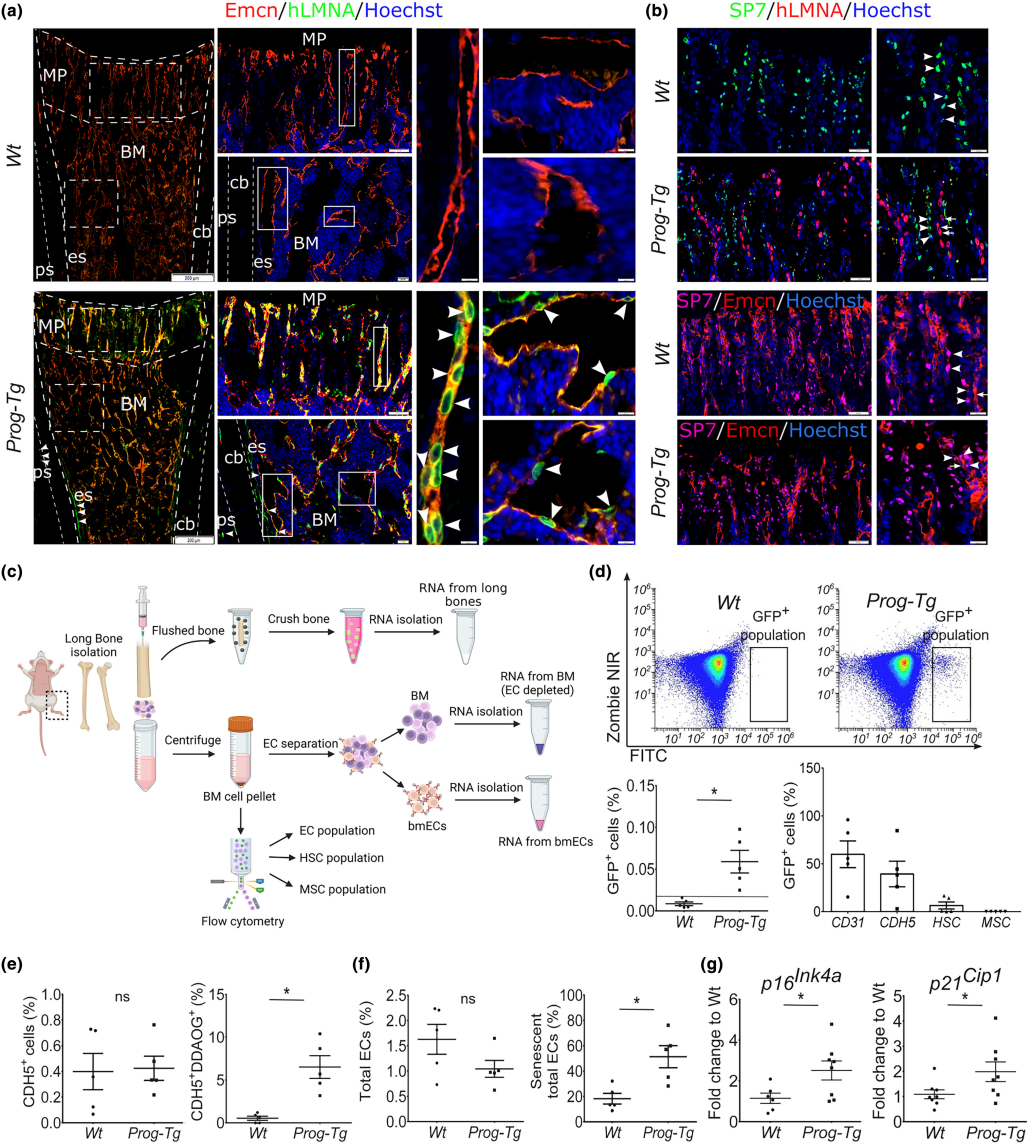
Specificity of progerin expression to vasculature in the bone of *Prog‐Tg* animals. (a) Representative immunofluorescence images of anti‐human lamin A staining (hLMNA) (arrowheads) with endomucin (Emcn)‐stained bone microvessels and (b) anti‐human lamin A staining (hLMNA) (arrows) with anti‐SP7 stained osteoprogenitors (arrowheads). Lower panel, SP7 (arrowheads) co‐stained with Emcn (arrows). Scale bars, 200 μm, 50 μm, 20 μm (young age ≤ 14 days). BM, bone marrow; cb, cortical bone; es, endosteum; MP, metaphysis; ps, periosteum. (c) Scheme depicting the workflow for the preparation of bone and BM extracts. (d) Representative flow cytometry scatter‐plots showing ZombieNIR^−^GFP^+^ cell population in the BM of *Prog‐Tg* but not *Wt* animals. Quantification of GFP^+^ cells in young *Wt* and *Prog‐Tg* animals (*n* = 5). (e, f) In vivo labeling of senescent BM cell populations using DDAOG far‐red probe followed by flow cytometry analysis. Quantification of (e) total CD45^−^Ter‐119^−^CDH5^+^ and senescent CD45^−^Ter‐119^−^CDH5^+^DDAOG^+^ (as proportion of total CD45^−^Ter‐119^−^CDH5^+^) and (f) total CD45^−^Ter‐119^−^CD31^+^ ECs and senescent CD45^−^Ter‐119^−^CD31^+^DDAOG^+^ (as proportion of CD45^−^Ter‐119^−^CD31^+^) in young animals (age ≤ 14 days, *n* = 5). (g) Gene expression levels of senescence markers in bone marrow‐derived endothelial cells (bmECs) (age ≤ 14, *n* = 6–9). Unpaired two‐tailed Student's *t*‐test (*p < 0.05, ns, not significant).

Our previous findings have demonstrated specific progerin expression in endothelial but not in non‐endothelial cell populations of the lung and cardiovascular tissues in *Prog‐Tg* mice leading to the accumulation of senescent cells (Manakanatas et al., 2022; Osmanagic‐Myers et al., 2019). To examine the endothelial specificity of progerin expression in the bone, we used anti‐human lamin A antibody (hLMNA) that specifically recognizes only human lamin A minigene (Figure S1) but not endogenous mouse lamin A (Osmanagic‐Myers et al., 2019; Sagelius et al., 2008). hLMNA stained nuclei were found specifically aligned within the Emcn‐stained microvasculature lining the periosteum and endosteum of the diaphysis as well as within the BM and metaphysis of *Prog‐Tg* mice while, as expected, completely absent in the vasculature of *Wt* mice, confirming endothelial‐specific expression in the bone (Figure 2a and Figure S2c). Importantly, hLMNA staining was completely absent from Sp7^+^ (osterix) stained cells indicating no leaky progerin expression in the osteoprogenitors (Figure 2b). Sp7^+^ cells, however, were found aligning in the vicinity of hLMNA^+^ nuclei as well as Emcn^+^ bone microvessels implying, similar to previous findings (Kusumbe et al., 2014), cross‐talk between these osteoprogenitors and the vasculature (Figure 2b). Since lamin A transgene is coupled to eGFP (Figure S1), we additionally analyzed GFP expression in freshly isolated BM using flow cytometry (Figure 2c). GFP^+^ cells were exclusively found in BM of *Prog‐Tg* but not *Wt* animals confirming specific expression of hLMNA transgene (Figure 2d). To determine the specificity of GFP expression, we analyzed GFP^+^ cell populations for the expression of known endothelial CD31^+^Cdh5^+^, mesenchymal CD45^−^PDGFRa^+^Sca‐1^+^, and hematopoietic Lin^−^Sca‐1^+^c‐Kit^+^ stem cell lineage markers using flow cytometry. Almost all GFP^+^ cells were found positive for endothelial markers (~59% CD31, ~39% Cdh5), no signals were detected for MSC markers, whereas a tiny proportion of around ~5% were found positive for HSC markers (Figure 2d). We reasoned that some signals might have been due to unspecific autofluorescence since flow cytometric analysis of the HSC population in young and adult animals revealed less than ~0.5% to be positive for Cdh5 (Figure S2d). Thus, we concluded that *Cdh5*‐driven expression of endothelial lineage marker is negligible in HSCs and is specific to EC populations of the BM confirming the EC‐specificity of *Cdh5*‐tTA driven mutated lamin A transgene (progerin) in *Prog‐Tg* mice.

We next examined the proportions of senescent endothelial cells in the bone marrow (bmECs) of *Prog‐Tg* mice in comparison to their *Wt* counterparts using an improved in vivo senescence‐associated beta‐galactosidase (SA‐β‐gal) activity assay which utilizes the far‐red 9H‐(1,3‐dichloro‐9,9‐dimethylacridin‐2‐one‐7‐yl) ß‐D galactoside (DDAOG) probe) (see Section 4). We first tested the specificity of DDAOG in vitro in ECs derived from *Wt* and *Prog‐Tg* mice using doxorubicin (dox)‐induced senescent *Wt*‐ECs as positive controls. Significantly increased DDAOG staining in *Prog‐Tg* and dox‐ECs in conjunction with elevated gene expression levels of senescence markers *p16* ^*Ink4a*^ and *p21* ^*Cip1*^ (Figure S2e,f) confirmed the specificity of DDAOG and senescence of lung *Prog‐Tg* ECs (Manakanatas et al., 2022). Importantly, flow cytometry of freshly isolated BM suspensions pre‐incubated with DDAOG, revealed significantly increased proportions (~10%) of senescent CDH5‐positive ECs (CD45^−^Ter‐119^−^CDH5^+^) with an increase up to ~50% in total CD31‐positive EC populations (CD45^−^Ter‐119^−^CD31^+^) whereas their respective total EC numbers remained unchanged (Figure 2e,f). Consistent with these findings, in ECs, isolated from the whole bone marrow (bmECs; Figure 2c), significantly increased gene expression levels of senescence markers (*p16* ^*Ink4a*^, *p21* ^*Cip1*^) were detected (Figure 2g). Altogether, these findings indicate that progerin expression is specifically confined to the vasculature leading to elevated endothelial senescence in the bone tissue.

### 2.3 Reduced osteogenesis in *Prog‐Tg* animals

**FIGURE 3:**
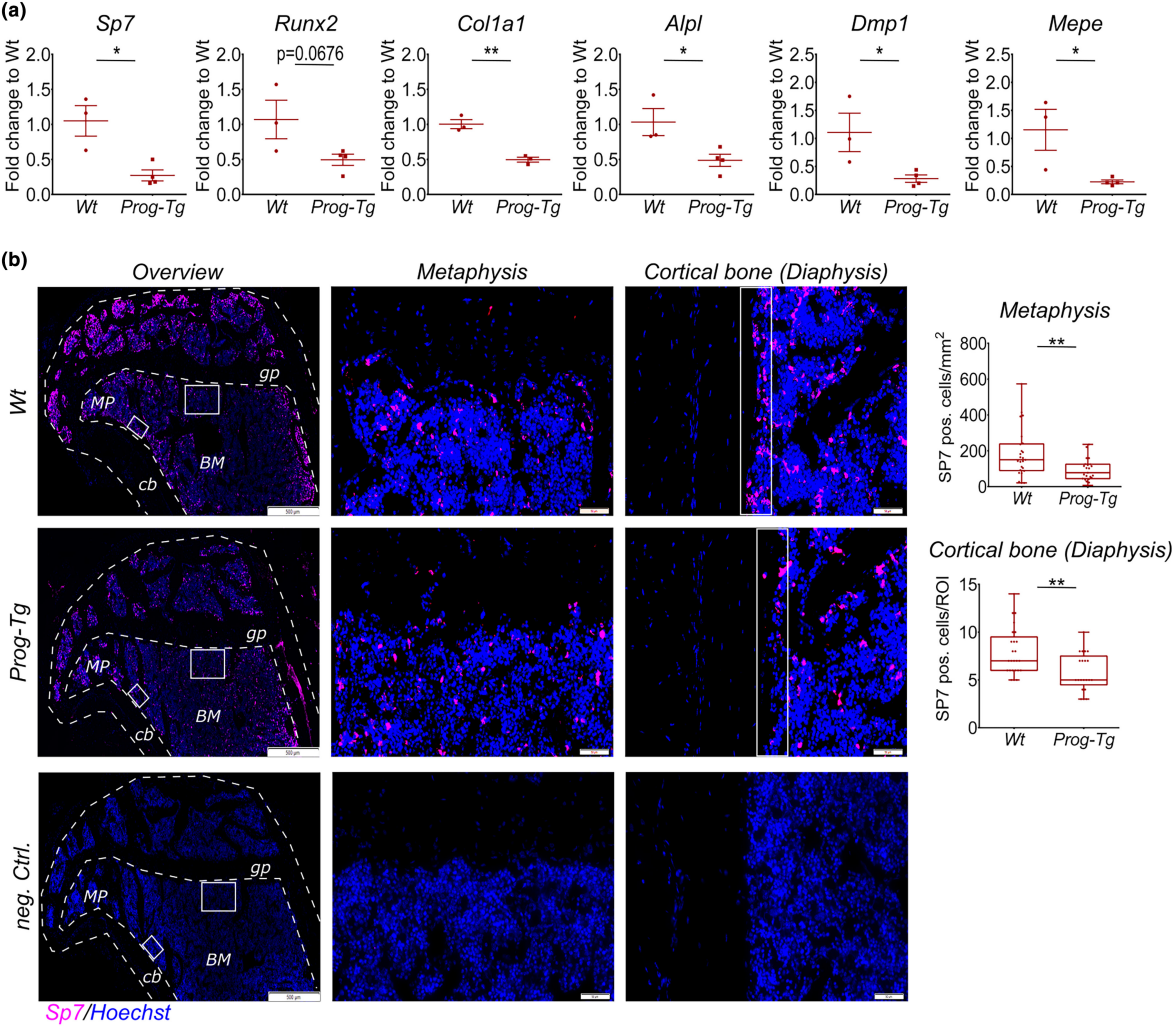
Reduced osteogenesis in *Prog‐Tg* animals. (a) Gene expression analysis of osteogenic markers in bone extracts from adult animals (age = 30–35 weeks, *n* = 3–4). Unpaired two‐tailed Student's *t*‐test (*p < 0.05, **p < 0.01). (b) Representative immunofluorescence images of Sp7^+^ osteoprogenitors and DNA‐stain Hoechst in tibia sections from adult *Wt* and *Prog‐Tg* animals. Negative controls show identical *Prog‐Tg* bone sections incubated with the secondary antibody and Hoechst. gp, growth plate; MP, metaphysis; BM, bone marrow; cb, cortical bone. Right panels, high power images of representative metaphysis and diaphysis regions used for quantification of Sp7^+^ cells. Each dot represents a number of SP7 positive cells per region of interest (ROI) (age = 30–35 weeks, *n* = 21–25 from 3 animals per genotype for each 3 different serial sections per animal analyzed). Scale bars, 500 μm and 100 μm. Data presented as median with boxes encompassing 25th to 75th percentile, and whiskers, minimum to maximum values. Mann Whitney *U* test (*p < 0.05, **p < 0.01).

To analyze if bone defects are rooted in changes in osteogenesis‐related genes, we performed gene expression analysis in long bone extracts separated from the whole BM of adult *Prog‐Tg* mice (Figure 2c). In bone extracts but not BM, we found a significantly reduced gene expression of a key transcription factor required for osteoblast differentiation, *Sp7* (osterix), and a tendency to lower levels of the transcriptional regulator, runt‐related transcription factor 2 (*Runx2*) (Figure 3a and Figure S3a). This was accompanied by a significant reduction in downstream targets of *Sp7* required for bone matrix formation, collagen type1a1 (*Col1a1*), and alkaline phosphatase (*Alpl*) in bone but not BM (Figure 3a and Figure S3a). Finally, a significant reduction in osteocyte markers, dentin‐matrix protein 1 (*Dmp1*), and matrix extracellular phosphoglycoprotein (*Mepe*) altogether implicated severely compromised differentiation to osteogenic lineage in *Prog‐Tg* mice (Figure 3a). Consistent with these observations, we found significantly reduced numbers of Sp7^+^ osteoprogenitors in trabecular (metaphysis) sections and regions lining the cortical regions of the diaphysis (Figure 3b).

On the contrary, unaltered levels of bone resorption regulating osteoclast‐specific factors, cathepsin K (*Ctsk*), nuclear factor of activated T cells (*Nfatc1*), receptor of NF‐κB ligand (*Rankl*) and osteoclastogenesis inhibitory factor osteoprotegerin (*Opg*) as well as tartrate‐resistant acid phosphatase (TRAP) staining of osteoclast fractions indicated no changes in osteoclasts (Figure S3b,c). Consistent with these findings, ex vivo osteoclastogenesis using HSC populations derived from *Prog‐Tg* and *Wt* mice revealed no significant changes in the number and size of differentiated osteoclast populations indicating unaffected osteoclast differentiation capacity of HSCs in *Prog‐Tg* mice (Figure S3d). Altogether, these findings supported the above notion that osteogenesis defects in *Prog‐Tg* animals were primarily rooted in impaired bone formation rather than bone resorption.

### 2.4 Long‐term reduction in differentiation capacity of *Prog‐Tg* ‐derived BMSCs caused by Wnt‐axis repression

**FIGURE 4:**
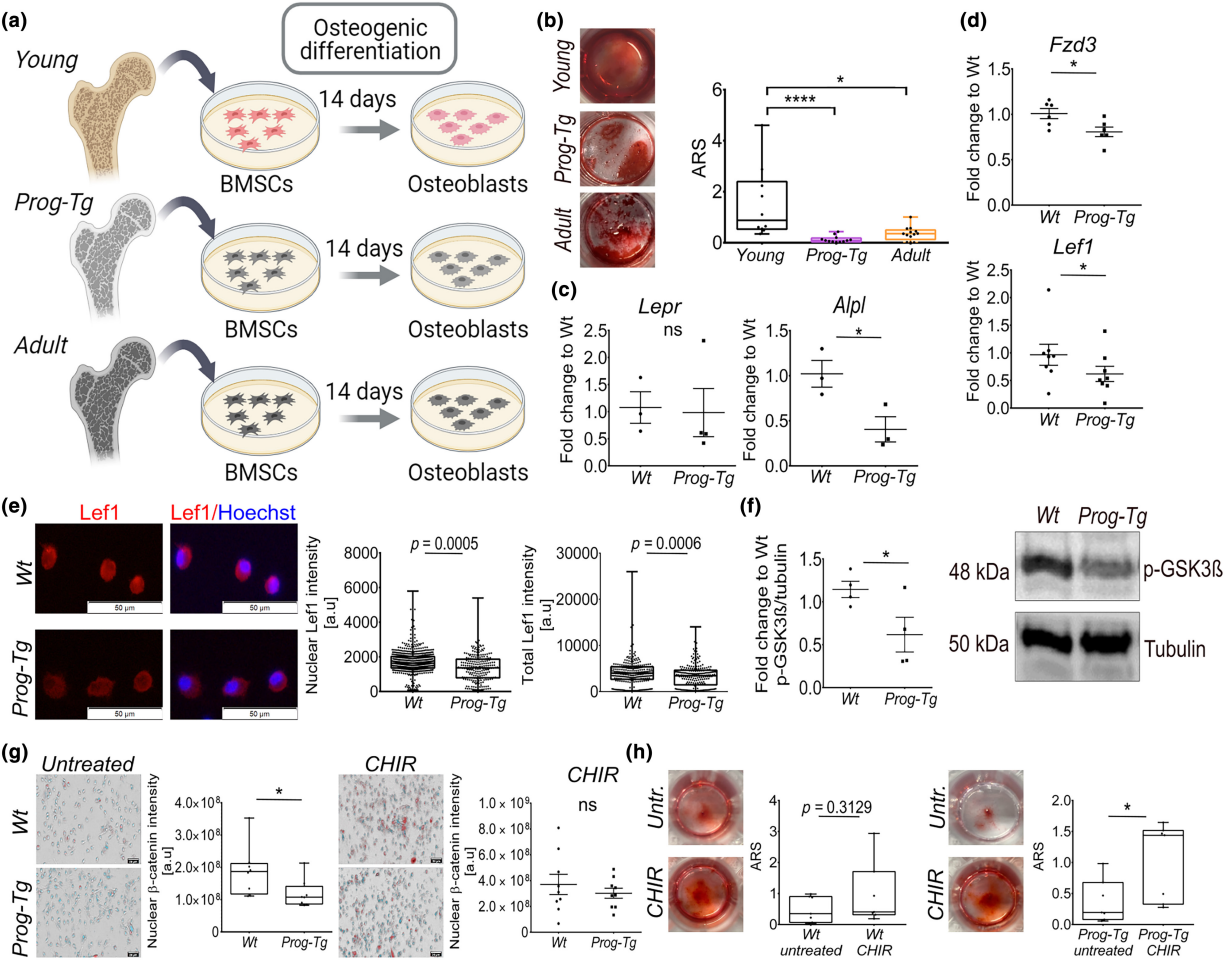
Diminished osteogenic differentiation and repression of Wnt signaling in BMSCs derived from the aged‐vascular niche. (a) Schematic illustration depicting the workflow for BMSC isolation from the BM niche of *Wt*, *Prog‐Tg*, and adult animals with subsequent osteogenic differentiation. (b) Representative images of Alizarin Red S (ARS) stained deposited mineralized matrix with the corresponding quantification (age ≤ 14 days, *n* = 10 *Wt*, *n* = 12 *Prog‐Tg* animals; age ~ 5–22 months, *n* = 13 adult *Wt* animals). Kruskal‐Wallis test with post hoc Dunn's multiple comparison test (*p < 0.05, ****p < 0.0001). (c) Gene expression analysis for *Alpl* and *Lepr* after 14 days of osteoblast differentiation (*n* = 3–4). (d) Gene expression levels of *Fzd3* and *Lef1* in BMSCs isolated from *Wt* and *Prog‐Tg* animals (age ≤ 14 days, *n* = 6 for *Fzd3* and *n* = 8 for *Lef1*). (e) Immunofluorescence images of BMSCs derived from *Wt* and *Prog‐Tg* animals stained with Lef1‐antibody and Hoechst. Quantification of total and nuclear Lef1 signal intensities. Each dot represents a cell (*n* = 275 cells for *Wt*, *n* = 220 cells for *Prog‐Tg*). Scale bar, 50 μm. (f) Quantitative immunoblot analysis of phospho‐GSK3β in long bone extracts from *Wt* and *Prog‐Tg* animals. Representative Western blots detecting anti‐phospho‐GSK3β (Ser9) and anti‐tubulin as loading control, run in parallel with the same samples on a separate gel (age ~ 20 weeks, *n* = 4). (g) Representative image of β‐catenin using transmission light microscopy combined with immunofluorescence staining of β‐catenin and Hoechst in BMSCs derived from *Wt* and *Prog‐Tg* animals either untreated or treated with the Wnt‐activator CHIR99021 for 24 h. High‐throughput quantitation of nuclear β‐catenin shown as average mean fluorescence intensity values (age ~ 20 weeks, *n* = 9 with 3 biological replicates in 3 technical replicates). Scale bar, 20 μm. (h) Representative images of ARS‐stained deposited mineralized matrix with the corresponding quantification of BMSCs derived from *Wt* and *Prog‐Tg* animals after 14‐day osteogenic differentiation in the absence (DMSO, untreated (Untr.)) or presence of CHIR99021 (CHIR) (*n* = 7). (b, e, g, h) Data presented as median with boxes encompassing 25th to 75th percentile, and whiskers, minimum to maximum values. (e, g, h) Mann Whitney *U* test (*p < 0.05). (c, d, f) Unpaired two‐tailed Student's *t*‐test (*p < 0.05, ns, not significant).

To test if the osteogenesis defects were rooted in impaired osteogenic differentiation of bone marrow stem cells (BMSCs), we conducted ex vivo differentiation of BMSCs toward osteoblast lineages. For this, we isolated BMSCs from the BM of young *Wt* and *Prog‐Tg* mice including adult *Wt* animals to test osteogenic differentiation in physiological aging (Figure 4a). Phenotypic enrichment for BMSC populations was verified by flow cytometry and by immunofluorescence microscopy using a common BMSC marker, leptin receptor (Lepr) (Matsuzaki et al., 2014) (Figure S4a,b). Indeed, significantly lower osteogenic differentiation capacity of BMSCs derived from young *Prog‐Tg* animals was detected using Alizarin Red S (ARS)‐staining of mineralized matrix deposits formed after 14‐day differentiation (Figure 4b and Figure S4c). The decline in the osteogenic potential of BMSCs from young *Prog‐Tg* mice was in the range of that observed in adult *Wt* animals implicating a substantial contribution of the aged vasculature on young BMSCs comparable to that observed in adult animals (Figure 4b). Consistent with paracrine vascular effects, no progerin expression (hLMNA) was detected in BMSCs derived from *Prog‐Tg* animals in contrast to evident staining in ECs (Figure S4b). This key finding, we additionally repeated for BMSCs derived from *LA‐Tg* and corresponding littermate *LM_LA‐Tg* control animals. We did not detect significant differences in the differentiation capacity of BMSCs derived from *LA‐Tg* control animals and those from respective *LM_LA‐Tg* littermates corroborating the progerin‐specific endothelial aging effect on osteoblast differentiation of BMSCs (Figure S4d). Moreover, differentiated osteogenic lineages from *Prog‐Tg* mice showed lower gene expression levels of bone formation marker alkaline phosphatase (*Alpl*) with no significant change in the levels of BMSC marker *Lepr* gene corroborating the above findings of lower osteogenic potential of BMSCs but no changes in their total pool (Figure 4c). This phenotype was not caused by changes in the clonogenicity of BMSCs, since no differences in the ability to generate colonies of fibroblast‐like cells using colony‐forming units‐fibroblast assay (CFU‐F) were detected in BMSCs derived from *Prog‐Tg*, also not *LA‐Tg* mice, compared to respective *Wt* and *LM_LA‐Tg* littermates (Figure S4e). Finally, it appeared not to be a consequence of increased apoptosis or necrosis of differentiated osteogenic populations, since no changes were detected in annexin V‐complementary NanoBIT luciferase assay with profluorescent DNA dye (Figure S4f). Thus, we concluded that BMSC differentiation defects are not caused by a diminished BMSC pool nor by increased apoptosis in *Prog‐Tg* animals but rather corroborate the above findings of persistent “memory” effects of the aged‐vascular endothelial niche on the osteogenic differentiation potential.

Impaired osteogenesis during HGPS aging but also physiological aging is mostly associated with a decline in Wnt‐signaling axis (Choi et al., 2018; Hernandez et al., 2010; Hofmann et al., 2014). Thus, to examine if Wnt‐repression is the cause of impaired osteogenesis in BMSCs derived from *Prog‐Tg* mice, we assessed gene expression levels of two key components of the Wnt‐signaling pathway, frizzled class receptor 3 (*Fzd3*) and lymphoid enhancer factor 1 (*Lef1*). Indeed, a significant downregulation of both *Fzd3* and *Lef1* pointed toward general repression of Wnt‐signaling in BMSCs from *Prog‐Tg* mice (Figure 4d). To validate these key findings, we included additional gene expression analyses in BMSCs from control *LA‐Tg* animals. We found no significant differences in gene expression levels of *Fzd3* and *Lef1* in BMSCs isolated from *LA‐Tg* animals compared to their *LM_LA‐Tg* littermates excluding the possibility that transgenic endothelial expression of wild‐type lamin A per se exerts an effect on the Wnt‐signaling axis in BMSCs (Figure S4g). Moreover, using immunofluorescence microscopy, we detected significantly lower Lef1 signals in the nuclei as well as in the total area of BMSCs derived from *Prog‐Tg* animals consistent with reduced gene expression level of *Lef1* (Figure 4e). Activation of canonical‐Wnt signaling is associated with the inhibition of the activity of glycogen synthase 3β (GSK3β) and consequently increased β‐catenin translocation to the nucleus (Choi et al., 2018; Galli et al., 2012). We detected significantly lower inhibitory phosphorylation of GSK3β (pSer9) in bone extracts from *Prog‐Tg* compared to *Wt* mice indicating enhanced GSK3β activity and thus, reduced Wnt‐signaling activity in vivo (Figure 4f). Therefore, we next performed high‐throughput image analysis to assess changes in β‐catenin nuclear localization. Significantly lower nuclear localization signals of β‐catenin in BMSCs derived from *Prog‐Tg* compared to *Wt* animals corroborated diminished Wnt‐activity (Figure 4g; untreated). Consistent with elevated GSK‐3β activity in *Prog‐Tg* mice, treatment with a potent Wnt‐activator selectively inhibiting GSK‐3β kinase, CHIR99021 (CHIR), increased the nuclear β‐catenin localization in *Prog‐Tg* as well as *Wt*‐derived BMSCs abolishing their differences observed prior treatment (Figure 4g; CHIR). Consistent with this observation continuous administration of CHIR during osteogenic differentiation boosted the osteogenic potential of *Prog‐Tg*‐derived BMSCs implying that their low osteogenic differentiation potential is rooted in Wnt‐signaling repression (Figure 4h).

### 2.5 Aged‐vascular niche deteriorates bone marrow microenvironment and BMSCs

**FIGURE 5:**
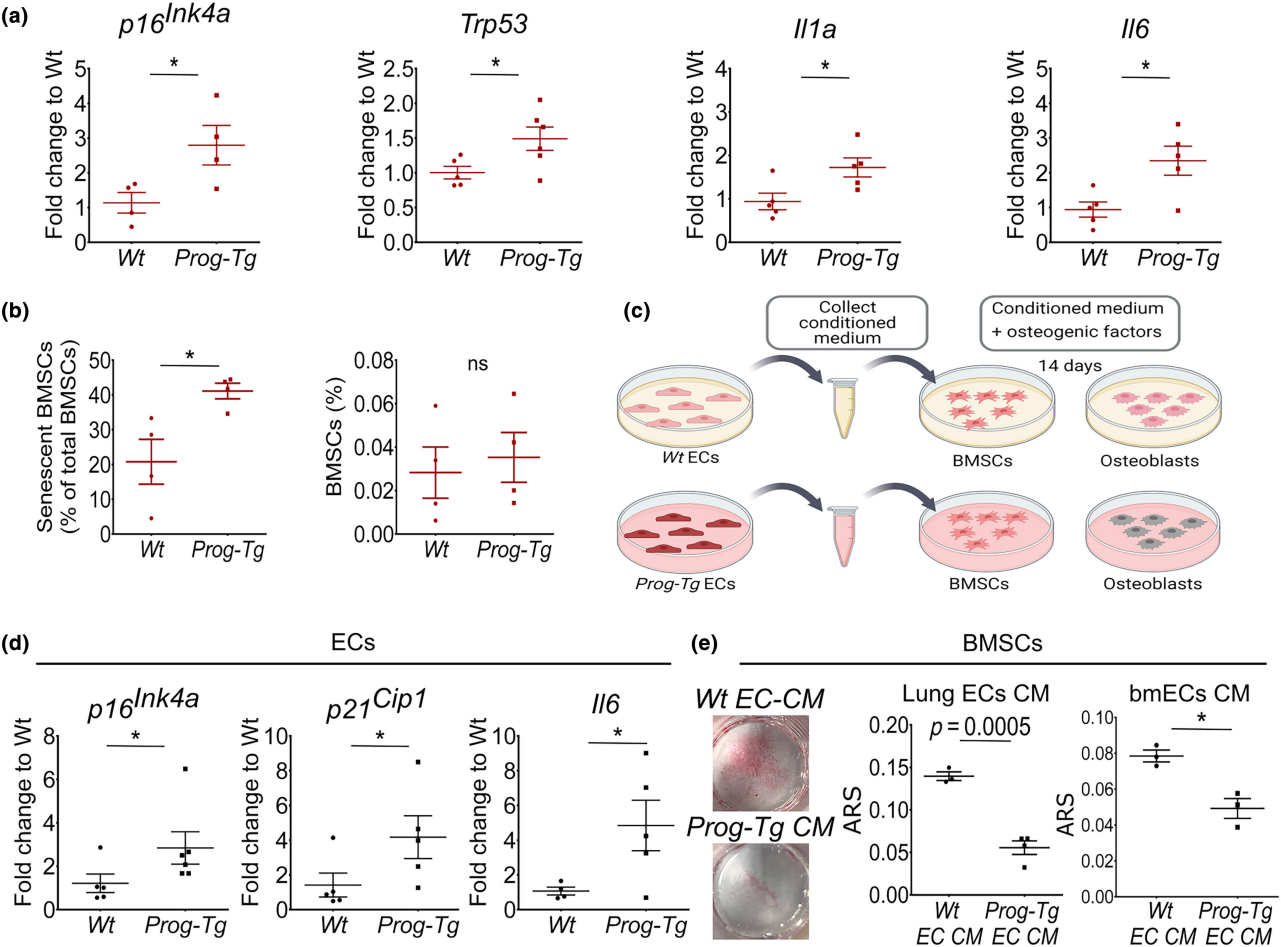
SASP factors of the aged‐vascular niche impair osteogenic differentiation of BMSCs. (a) Gene expression levels of depicted genes in extracts from EC‐depleted bone marrow (BM) (age = 30–35 weeks, *n* = 4–6). (b) Quantification of CD45^−^Sca‐1^+^PDGFRα^+^ BMSCs and senescent CD45^−^Sca‐1^+^PDGFRα^+^DDAOG^+^ BMSCs (as proportion of total CD45^−^Sca‐1^+^PDGFRα^+^ BMSCs) in the BM of adult *Wt* and *Prog‐Tg* animals analyzed by DDAOG far‐red probe followed by flow cytometry analysis (age = 30–35 weeks, *n* = 4). (c) Scheme for osteogenic differentiation of young *Wt* BMSCs (age ≤ 14 days) in the presence of conditioned media (CM) collected from young *Wt* and *Prog‐Tg* EC cultures. (d) Characterization of ECs derived from *Wt* and *Prog‐Tg* animals from which CM was collected (age ≤ 14 days, *n* = 5–6). (e) Representative images and quantification of ARS‐stained matrix deposits after differentiation of young BMSCs in the presence of CM from *Wt* and *Prog‐Tg* ECs isolated from lung (Lung ECs CM) or BM (bmECs CM) (*n* = 3–4). Unpaired two‐tailed Student's *t*‐test (*p < 0.05, ns, not significant).

Aged niches exert negative extrinsic effects on surrounding stem cells (Brunet et al., 2022), however, the specific paracrine effects of an aged‐vascular niche on BMSCs and its microenvironment in “in vivo” setting are hitherto unknown. To discern paracrine changes introduced by the aged‐vascular niche in the BM and bone, we assessed gene expression levels of senescence and SASP markers in the BM separated from bmECs and in bone extracts (Figure 2c). Significant upregulation of senescence markers *p16* ^*Ink4a*^ and *Trp53* (but not *p21* ^*Cip1*^) including also inflammatory SASP markers (*Il1a*, *Il6*) was detected in the BM microenvironment of adult *Prog‐Tg* animals compared to *Wt* littermates (Figure 5a and Figure S5a). Paracrine senescence appeared not to affect bone per se since no change in senescence markers was detected in BM‐depleted bone extracts (Figure S5b). These findings hinted profound paracrine effects on BM microenvironment through the senescent vascular endothelial niche.

Repression of Wnt‐signaling has recently emerged as a key contributing factor to senescence development in BMSCs (Lehmann et al., 2022). To determine if BMSC populations are affected by elevated cellular senescence of the BM we performed in vivo DDAOG staining of SA‐β‐Gal activity in BM from adult mice followed by flow cytometric immunophenotypic analysis of CD45^−^Sca‐1^+^PDGFR^+^ BMSCs. Indeed, we found significantly increased proportions of senescent DDAOG^+^BMSCs in adult *Prog‐Tg* mice compared to those derived from their *Wt* littermates (Figure 5b). No change in their total numbers was observed either by using flow cytometry or Lepr staining of BMSC in whole bone sections confirming our earlier results of unchanged BMSC pool in *Prog‐Tg* mice (Figure 5b and Figure S5c). However, BMSCs derived from young *Prog‐Tg* mice did not show elevated senescence (Figure S5d), which is consistent with unchanged clonogenicity (see Figure S4e). Altogether, we reasoned that BMSC senescence manifests at later age stages in *Prog‐Tg* mice in contrast to early occurring repressive Wnt‐signals perturbing osteogenesis.

To directly assess these paracrine repressive effects, we tested the impact of *Prog‐Tg* EC‐derived conditioned media (CM) on the osteogenic potential of *Wt*‐derived BMSCs (Figure 5c). Conditioned media were collected from primary ECs isolated from lung tissues of *Prog‐Tg* and *Wt* animals. The latter were subsequently tested for gene expression levels of senescence and SASP markers as well as EC purity using immunofluorescence microscopy analysis of endothelial‐specific cell–cell junction marker, PECAM‐1 (CD31). Consistent with our earlier shown results (see Figure S2e,f), *Prog‐Tg* ECs, used for collection of CM, showed increased gene expression levels of senescence and SASP markers, *p16* ^*In4a*^, *p21* ^*Cip1*^ and *Il6* (Figure 5d) and similar to *Wt*‐ECs high level of EC purity (Figure S5e; left). Next, we assessed endothelial paracrine effects on BMSCs isolated from young *Wt* animals by testing their osteogenic capacity in the presence of corresponding EC‐derived CM. Indeed, young *Wt*‐derived BMSCs showed diminished osteogenic differentiation in the presence of CM derived from senescent *Prog‐Tg* ECs as demonstrated by significantly reduced ARS‐stained mineralized matrix deposits (Figure 5e, Lung ECs CM). This was accompanied by trends towards elevated SASP markers *Il6*, *Il1a*, and *Trp53* (Figure S5f). We next repeated these findings using bmECs that needed to be cultured in mild hypoxia (~10% O_2_) in order to obtain sufficient yields for CM collection. Similar to lung‐derived ECs, CMs of bmECs had a significant inhibitory effect on the osteogenic potential of *Wt*‐derived BMSCs confirming systemic paracrine repressive signals of the aged vasculature on the bone (Figure 5e, bmECs CM; Figure S5e).

### 2.6 Paracrine miR‐31‐mediated Wnt‐axis repression inhibits BMSC osteogenesis

**FIGURE 6:**
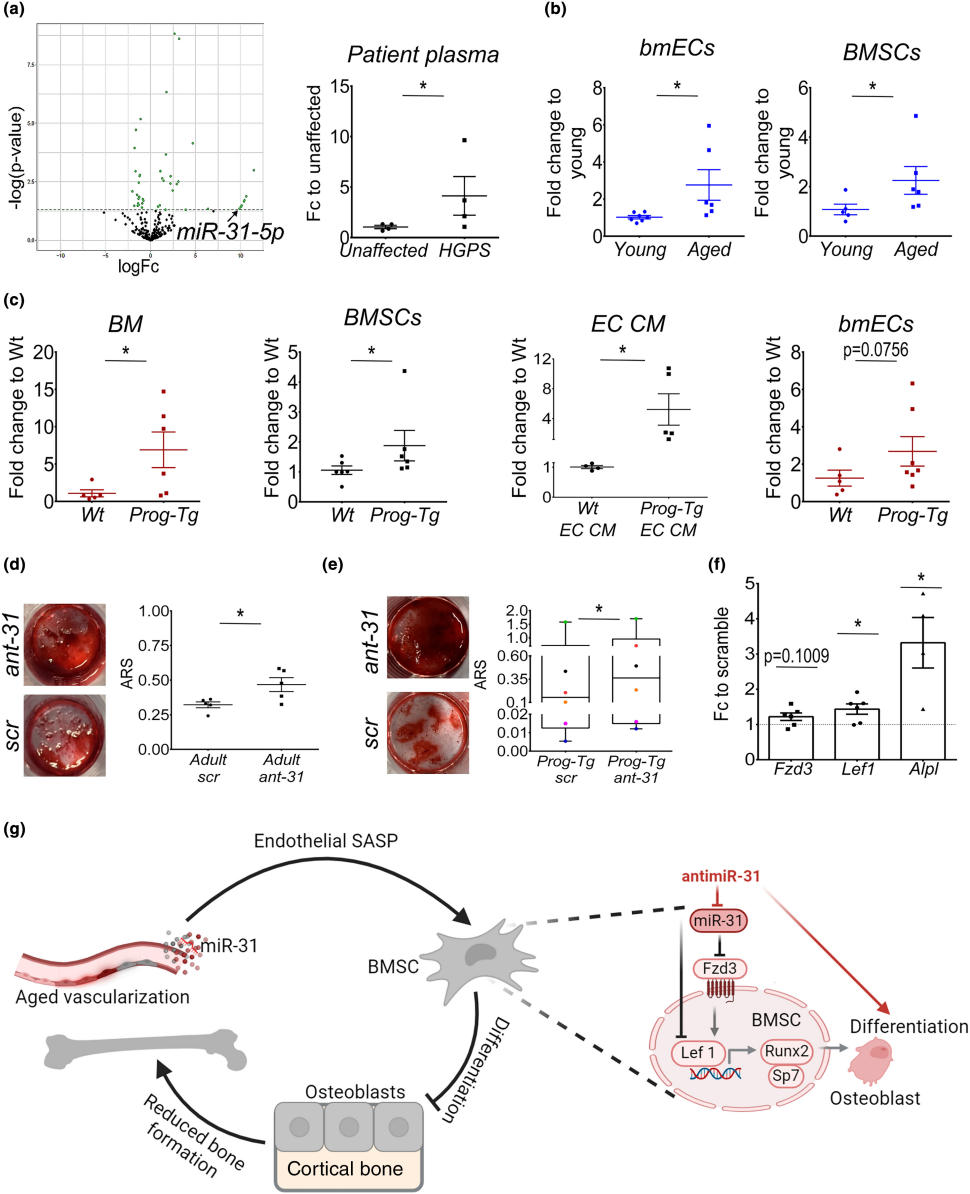
Inhibition of miR‐31‐5p boosts osteogenesis of BMSCs from the aged‐vascular niche through de‐repression of Wnt‐axis. (a) Volcano plot depicting differential expression analysis of miRs in plasma from HGPS patients (*n* = 3) and age‐ and gender‐matched controls (*n* = 3) using depicted thresholds with Benjamini‐Hochberg adjusted *p*‐values. Gene expression levels of miR‐31‐5p in plasma from HGPS patients and unaffected controls (*n* = 4). (b) miR‐31‐5p gene expression levels in bmECs and enriched BMSCs derived from Wt animals (young ~3 months and aged ~22 months, *n* = 5–7) and (c) EC‐depleted BM (BM), BMSCs, extracellular vesicles derived from EC‐conditioned media (EC CM) and bmECs derived from *Wt* and *Prog‐Tg* animals (age ≤ 14 days (black color), age = 30–35 weeks (red color), *n* = 4–7). (d, e) Osteogenic differentiation of scramble (scr) and antimiR31‐5p (ant‐31) treated (d) adult BMSCs and (e) *Prog‐Tg* derived BMSCs with quantification of ARS‐stained deposits (age ~ 5–22 months for adult, age ≤ 14 days for *Prog‐Tg*; *n* = 5–6). (f) Gene expression analysis of *Fzd3*, *Lef1*, and *Alpl* shown as fold change to corresponding scramble control (age ≤ 14 days, *n* = 4–6). (b, c) Unpaired two‐tailed Student's *t*‐test (*p < 0.05, ns, not significant). (d) Paired Student's t‐test (antimiR‐31‐5p treated vs. scramble, *p < 0.05). (e, f) Wilcoxon Signed Rank test (antimiR‐31‐5p treated vs. scramble, *p < 0.05). (g) Proposed model depicting miR‐31‐mediated paracrine Wnt‐repressive signals derived from aged‐vascular niche hinder osteogenic differentiation capacity of BMSCs. Inhibition of miR‐31‐5p (antimiR‐31) boosts osteogenesis in BMSCs through de‐repression of Fzd3/Lef1‐levels. Senescent and paracrine senescent ECs marked in grey and darker red colors.

We next set out to find relevant endothelial factors that may exert long‐term persistent repression of Wnt‐signaling. Emerging data points to the role of microRNAs (miRs) in controlling gene expression, with circulatory miRs such as endothelial‐derived SA‐miRs we previously identified, having the capacity to be uptaken by surrounding cells and act in a paracrine repressive fashion (Grillari et al., 2021; Manakanatas et al., 2022). To screen for potential Wnt‐repressive miR candidates physiologically relevant for HGPS patients and the aging population, we first performed miR‐profiling in the plasma of HGPS patients and corresponding unaffected controls (kindly provided by Progeria Research Foundation). We narrowed down endothelium‐derived miR candidates from HGPS patient plasma by subsequent comparison with previously identified differentially expressed miRs in respective plasma and EC samples from *Prog‐Tg* mice (Manakanatas et al., 2022). Forty‐seven differentially expressed miRs were found in human HGPS patient plasma among which miR‐31‐5p was found concomitantly elevated in plasma and ECs from *Prog‐Tg* mice as well (Figures 6a and Figure S6a,b). This together with previous reports on the biomarker role of miR‐31‐5p in age‐related bone disease (Heilmeier et al., 2022; Weigl et al., 2021) implicated potential endothelial‐specific paracrine function on bone physiologically relevant to HGPS patients and potentially elderly as well. Upregulation of miR‐31‐5p could additionally be validated by qPCR analysis performed in plasma from HGPS patients and unaffected controls confirming the significance of this finding (Figure 6a, right). Moreover, we found significant upregulation of miR‐31‐5p in bone marrow ECs (bmECs) as well as BMSCs derived from BM of aged mice (~22 months) corroborating high importance of this miR in physiological aging as well (Figure 6b).

Furthermore, in *Prog‐Tg* mice significant elevation of miR‐31‐5p in the BM microenvironment, BMSC populations, and extracellular vesicles enriched from EC‐conditioned media with high trends in bmECs implicated predominantly active secretion of this miR and paracrine function (Figure 6c). Importantly, in BMSCs and plasma from *LA‐Tg* control animals, miR‐31‐5p expression levels remained unchanged confirming progerin‐specific effect (Figure S6c and for plasma [Manakanatas et al., 2022]).

Notably, we found miR‐31‐5p to have the capacity of Wnt‐signaling repression since several Wnt‐signaling components are either strongly predicted and/or experimentally confirmed mRNA targets according to TargetScanMouse, version 8.0 (Lewis et al., 2005). Among Wnt‐component targets, *Fzd3* was found at the top mRNA target list with four 3′UTR binding sites for miR‐31‐5p strongly suggesting its role in mediating *Fzd3* repression in BMSCs from *Prog‐Tg* mice (Figure S6d). To test if miR‐31‐5p has the potential to exert paracrine effects on osteogenic differentiation of BMSCs, we tested the effects of a selective antimiR‐31‐5p inhibitor (ant‐31; Figure S6e). Indeed, antimiR‐31‐5p boosted osteogenic differentiation potential of BMSCs derived from adult but not young *Wt* animals highlighting miR‐31‐5p repressive effects on osteogenesis in physiological aging as well (Figure 6d and Figure S6f,g). Consistent with this, similar effects were observed in BMSCs derived from young *Prog‐Tg* animals (Figure 6e and Figure S6g). We noticed some variations between treatments in BMSCs from different mouse litters of *Prog‐Tg* mice (marked by the same color in Figure 6e). This may be explained by a known increase in variability associated with senescent cells and their paracrine effects in aging (Wiley et al., 2017). Importantly, we found significantly elevated gene expression levels of osteogenic marker *Alpl* paralleled by de‐repression of Wnt‐targets, *Fzd3*, and *Lef1*, corroborating osteogenesis repressive function of miR‐31‐5p with inhibitory effects on Wnt‐signaling (Figure 6f). In summary, our findings indicate that miR‐31‐5p significantly contributes to the vascular‐mediated osteogenic decline of BMSCs in aging operating through paracrine repression of Wnt‐signaling (Figure 6g).

## 3 DISCUSSION

Aging has been shown to have deteriorating effects on the BM niche affecting hematopoiesis and stem cell fate decision (Biswas et al., 2023; Brunet et al., 2022). Hitherto, these effects were described in the context of cumulative aging of BM stem cell niches describing mainly effects on HSCs (Biswas et al., 2023; Brunet et al., 2022; Ho et al., 2019). To specifically study the effects of the aged‐vascular niche we used an endothelial HGPS aging mouse model (*Prog‐Tg*) (Manakanatas et al., 2022; Osmanagic‐Myers et al., 2019) with elevated cellular senescence in the bone vasculature as demonstrated in this study. Here, we show that aged vasculature induces phenotypical age‐related changes associated with cortical thinning and low bone volume fraction in line with the development of cellular senescence and SASP in the BM niche resembling some features of osteoporosis (Doolittle et al., 2021). Mechanistically, we demonstrate that this phenotype is caused by long‐lasting inhibitory signals of the aged vasculature on osteogenic differentiation of BMSCs acting through repression of Wnt‐signaling.

A hallmark of aging including HGPS premature aging‐related changes in bone structure is the loss of bone mass and thinning of the cortex (Cabral et al., 2023; Ferguson et al., 2003; Whitmarsh et al., 2019). Similar bone loss and cortical thinning outcomes observed in our endothelial progeria aging mouse model highlight the physiological significance of our findings in aging per se demonstrating for the first time a clear role of endothelial aging in bone homeostasis. Previous reports have shown vascular network rarefication in aging (Grunewald et al., 2021). Although we found reduced vascular networks in adult *Prog‐Tg* animals, no changes were observed in the vasculature of young animals with still evident osteogenesis defects, excluding the possibility that the latter were caused by capillary rarefication. However, since angiogenesis is coupled with osteogenesis (Kusumbe et al., 2014) we cannot rule out that with aging, osteogenesis defects contribute to reduced vascularization in adult animals and vice versa. Nevertheless, osteogenesis defects detected at a very early age corroborate our findings of inhibitory vascular endothelial paracrine signals as their primary cause. The regulation of bone homeostasis involves a fine‐tuned action of gene networks requiring activation of early and late transcription factors that in turn lead to increased gene expression of extracellular matrix components (Galli et al., 2012). In adult *Prog‐Tg* animals significantly reduced gene expression of early transcription factors *Runx2* and *Sp7* concomitant with diminished levels of downstream targets, *Alpl* and *Col1a1*, point towards an osteogenesis defect already very early during differentiation. In line with these findings, the Sp7^+^ osteoprogenitor pool in *Prog‐Tg* animals is significantly reduced. Thus, it is conceivable that osteocytes developing from these osteoprogenitors are also found in lower numbers as demonstrated by diminished gene expression levels of osteocyte‐specific genes and significantly increased empty osteocyte lacunae detected already in young animals. These findings implicate that the aged‐vascular niche contributes at the very early stage to empty osteocyte lacunae characteristic for age‐related bone loss (Hemmatian et al., 2017). Similar osteogenesis defects were shown by Schmidt et al. (Schmidt et al., 2012) using tissue‐specific progerin expression in Sp7^+^ osteoprogenitors highlighting intrinsic osteogenesis defects. In our study, clear absence of lamin A transgene expression in Sp7^+^ osteoprogenitors and BMSCs from *Prog‐Tg* animals in contrast to strong signals in ECs rules out the possibility of leaky progerin expression consistent with our previous findings (Osmanagic‐Myers et al., 2019). Thus, these data in addition to the in vitro findings using conditioned media, clearly point toward osteogenesis defects rooted in EC‐derived paracrine effects.

Cortex thinning may be caused by impaired bone formation but also by increased bone resorption and osteoclastogenesis (Doolittle et al., 2021). Since we observe no changes in osteoclastogenesis, numbers of TRAP^+^ osteoclasts in tissues, and gene expression levels of osteoclast‐related markers, we do not consider that there are significant effects on bone resorption and conclude that paracrine effects of the aged‐endothelium have a predominant role in bone formation. The decline of osteogenic capacity in BMSCs in aging is associated with increased skewing toward an adipogenic lineage that contributes to age and menopause‐associated BM adiposity (Wang et al., 2022). Histological analysis of BM sections from *Prog‐Tg* animals showed no signs of increased adiposity indicating that reduced osteogenic differentiation of BMSCs is not compensated by increased adipogenesis highlighting endothelial‐specific aging effects. Furthermore, flow cytometry analysis of BMSC subpopulations derived from *Prog‐Tg* in conjunction with analysis of Lepr^+^ BMSCs show no quantitative changes in BMSCs that would indicate stem cell depletion, a phenomenon frequently observed in aging (Brunet et al., 2022).

Our model supports vascular effects acting predominantly on the cortical bone which is also consistent with previous findings showing that VEGF “rejuvenated” vasculature rescues cortical thinning in aging (Grunewald et al., 2021). We speculate that such increased sensitivity of cortical regions to vascular changes may be associated with regional heterogeneity of vascular networks and/or changes in oxygen tension (Kusumbe et al., 2014). We do not consider the lack of evident trabecular changes a discrepancy since we show selective paracrine effects of aged vasculature on “healthy” or “not intrinsically aged” bone in vivo that may not necessarily coincide with all bone intrinsic age‐related changes.

Stable cell cycle arrest termed cellular senescence is an important hallmark of aging shown to significantly contribute to age‐related pathologies (Lopez‐Otin et al., 2023; Zhang et al., 2022). One key negative aspect of the presence of senescent cells is considered to be their altered communication with the microenvironment through the secretion of pro‐inflammatory cytokines, pro‐fibrotic factors, growth factors, and miRs, also termed senescence‐associated secretory phenotype (SASP) that exerts damaging effects on surrounding tissues (Acosta et al., 2013). Here, we demonstrate increased levels of senescence markers in ECs derived from the BM of *Prog‐Tg* mice which is, to our knowledge, the first report demonstrating in vivo development of selective senescence of the BM vascular niche during aging. Concomitantly, high levels of senescence markers *p16* ^*Ink4a*^, *Trp53* with prominent upregulation of inflammatory SASP factors *Il1a*, *Il6*, and *Tnfa* detected in the residual BM further strengthen our findings of deteriorating paracrine effects of the aged‐endothelium in the BM. However, it must be noted, that systemic effects cannot be excluded since premature aging in the whole‐body vasculature was introduced in *Prog‐Tg* mice.

Mechanistically, we show that the aged‐vascular niche induces repression of Wnt‐signaling in BMSCs demonstrated by corresponding changes in the major components of this signaling axis, *Fzd3*, GSK3β, β‐catenin, and *Lef1* (Figure 6g). Canonical‐Wnt signaling is initiated by the binding of secreted Wnt‐glycoproteins to transmembrane Fzd receptors. This in turn leads to the activation of intracellular signaling cascades associated with the inhibition of GSK3β and stabilized beta‐catenin complexes entering the nucleus and activating transcription factors TCF/Lef1 (Galli et al., 2012). Wnt‐input signals are a prerequisite for the process of osteoblastogenesis at various stages of differentiation with reports showing direct binding of TCF/Lef1 to promoter regions of early osteogenesis genes (Gaur et al., 2005). Wnt‐signaling pathway that has emerged as a key component of longevity pluripotency signaling networks is known to decline during physiological and HGPS aging, however, the causes of this repression are not fully clear (Choi et al., 2018; Hofmann et al., 2014; Meshorer & Gruenbaum, 2008). Here, we show by combining microRNA‐transcriptomics of plasma samples derived from HGPS patients with extensive analysis in endothelial progeria mice that the aged‐vascular niche acts through abundant Wnt‐repressive signals mediated by secretory miR‐31‐5p. Importantly, the finding of elevated miR‐31‐5p levels in HGPS patients highlights the physiological importance of this paracrine mediator. miR‐31‐5p was found elevated not just in the circulation but also dramatically in the BM microenvironment including BMSCs per se corroborating our model of systemic Wnt‐repression. Furthermore, we find elevated levels of miR‐31‐5p in bmECs and BMSCs from physiologically aged mice as well as antimiR‐31 boosting effects on the osteogenesis of BMSCs from adult *Wt* mice. The latter together with previous reports of miR‐31‐5p mediated osteogenesis inhibitory effects associated with osteoporosis in the elderly indicate that similar mechanisms operate in physiological aging as well (Weigl et al., 2021; Weilner et al., 2016; Xu et al., 2018).

Our findings demonstrate for the first time that elevated miR‐31‐signals originate mainly from the aged‐vascular niche acting in a paracrine long‐lasting Wnt‐repressive fashion on osteoprogenitors within the BM microenvironment.

In this regard, there is a growing body of evidence supporting the ability of miRs to exert long‐lasting non‐autonomous effects (reviewed in Grillari et al., 2021). This can be exemplified by the recently shown deteriorating effects of miRs secreted from the aged bone matrix that upon uptake by vascular smooth muscle cells cause the so‐called “calcification paradox” (Wang et al., 2022). The miR‐31 long‐term mode of action is most likely mediated through direct effects not just on the primary Wnt‐receptor *Fzd3* but also on *Lef1*, *a* key transcription factor that directly activates early osteogenesis through Runx2 (Gaur et al., 2005). Mechanistically, we clearly show that de‐repression of Wnt‐signaling, either through antmiR or direct Wnt‐activator CHIR, boosts the osteogenesis in BMSCs underpinning the key role of miR‐31‐mediated Wnt‐regulatory axis during bone formation (Figure 6g). In conclusion, we show for the first time in vivo that the aged BM vascular niche through paracrine repressive signals on the Wnt‐axis contributes to a functional decline of BMSCs leading to reduced osteogenesis and bone loss in aging. Our findings pave ground for microRNA‐based treatments of bone loss in HGPS patients as well as elderly.

## 4 EXPERIMENTAL PROCEDURES

Experimental procedures are described in detail in Appendix S1 accompanying this article.

## AUTHOR CONTRIBUTIONS

S.O‐M. conceived of the project and supervised its execution. S.O‐M. together with V.F. designed the experiments and wrote the manuscript. V.F. assembled the figures. V.F., F.M., M.B., I.F., K.K., and E.N. performed the experiments. Micro‐CT data were conceptualized by P.P. and conducted by K.W. Intellectual input was given by J.G. who has also conceptualized nanoCT experiments conducted by P.H. miRNA library preparation profiling was done by TAmiRNA, bioinformatics analysis by A.D. and formal analysis by M. Hackl. Scientific advice was provided by P.P., R.F., H.S., M. Hengstschläger. Resources were provided primarily by S.O‐M. and M. Hengstschläger, and also H.S. and R.F. All of the authors reviewed and edited the manuscript.

## FUNDING INFORMATION

This research was funded in whole, by the Austrian Science Fund (FWF) [P 32595‐B] to S.O‐M. For the purpose of open access, the author has applied a CC BY public copyright license to any Author Accepted Manuscript version arising from this submission.

## CONFLICT OF INTEREST STATEMENT

The authors declare that they have no conflicts of interest.

## Supporting information


Figure S1

Figure S2

Figure S3

Figure S4

Figure S5

Figure S6

Table S1

Appendix S1

## Data Availability

The data that support the outcomes of this study are available from the paper and supporting material. Upon request, raw data are available from the corresponding author.

## References

[acel14139-bib-0001] Acosta, J. C. , Banito, A. , Wuestefeld, T. , Georgilis, A. , Janich, P. , Morton, J. P. , Athineos, D. , Kang, T. W. , Lasitschka, F. , Andrulis, M. , Pascual, G. , Morris, K. J. , Khan, S. , Jin, H. , Dharmalingam, G. , Snijders, A. P. , Carroll, T. , Capper, D. , Pritchard, C. , … Gil, J. (2013). A complex secretory program orchestrated by the inflammasome controls paracrine senescence. Nature Cell Biology, 15, 978–990. 10.1038/NCB2784 23770676 PMC3732483

[acel14139-bib-0002] Biswas, L. , Chen, J. , De Angelis, J. , Singh, A. , Owen‐Woods, C. , Ding, Z. , Pujol, J. M. , Kumar, N. , Zeng, F. , Ramasamy, S. K. , & Kusumbe, A. P. (2023). Lymphatic vessels in bone support regeneration after injury. Cell, 186(2), 382–397e4. 10.1016/j.cell.2022.12.031 36669473

[acel14139-bib-0003] Brunet, A. , Goodell, M. A. , & Rando, T. A. (2022). Ageing and rejuvenation of tissue stem cells and their niches. Nature Reviews. Molecular Cell Biology, 24, 45–62. 10.1038/s41580-022-00510-w 35859206 PMC9879573

[acel14139-bib-0004] Cabral, W. A. , Stephan, C. , Terajima, M. , Thaivalappil, A. A. , Blanchard, O. , Tavarez, U. L. , Narisu, N. , Yan, T. , Wincovitch, S. M. , Taga, Y. , Yamauchi, M. , Kozloff, K. M. , Erdos, M. R. , & Collins, F. S. (2023). Bone dysplasia in Hutchinson‐Gilford progeria syndrome is associated with dysregulated differentiation and function of bone cell populations. Aging Cell, 22, e13903. 10.1111/acel.13903 37365004 PMC10497813

[acel14139-bib-0005] Cenni, V. , Capanni, C. , Mattioli, E. , Schena, E. , Squarzoni, S. , Bacalini, M. G. , Garagnani, P. , Salvioli, S. , Franceschi, C. , & Lattanzi, G. (2020). Lamin A involvement in ageing processes. Ageing Research Reviews, 62, 101073. 10.1016/j.arr.2020.101073 32446955

[acel14139-bib-0006] Chen, J. , Hendriks, M. , Chatzis, A. , Ramasamy, S. K. , & Kusumbe, A. P. (2020). Bone vasculature and bone marrow vascular niches in health and disease. Journal of Bone and Mineral Research, 35(11), 2103–2120. 10.1002/jbmr.4171 32845550

[acel14139-bib-0007] Choi, J. Y. , Lai, J. K. , Xiong, Z. M. , Ren, M. , Moorer, M. C. , Stains, J. P. , & Cao, K. (2018). Diminished canonical beta‐catenin signaling during osteoblast differentiation contributes to osteopenia in progeria. Journal of Bone and Mineral Research, 33(11), 2059–2070. 10.1002/jbmr.3549 30001457 PMC7739562

[acel14139-bib-0008] Doolittle, M. L. , Monroe, D. G. , Farr, J. N. , & Khosla, S. (2021). The role of senolytics in osteoporosis and other skeletal pathologies. Mechanisms of Ageing and Development, 199, 111565. 10.1016/j.mad.2021.111565 34499959 PMC8490322

[acel14139-bib-0009] Eriksson, M. , Brown, W. T. , Gordon, L. B. , Glynn, M. W. , Singer, J. , Scott, L. , Erdos, M. R. , Robbins, C. M. , Moses, T. Y. , Berglund, P. , Dutra, A. , Pak, E. , Durkin, S. , Csoka, A. B. , Boehnke, M. , Glover, T. W. , & Collins, F. S. (2003). Recurrent de novo point mutations in lamin A cause Hutchinson‐Gilford progeria syndrome [Research Support, Non‐U.S. Gov't]. Nature, 423(6937), 293–298. 10.1038/nature01629 12714972 PMC10540076

[acel14139-bib-0010] Ferguson, V. L. , Ayers, R. A. , Bateman, T. A. , & Simske, S. J. (2003). Bone development and age‐related bone loss in male C57BL/6J mice. Bone, 33, 387–398. 10.1016/S8756-3282(03)00199-6 13678781

[acel14139-bib-0011] Galli, C. , Piemontese, M. , Lumetti, S. , Manfredi, E. , Macaluso, G. M. , & Passeri, G. (2012). The importance of WNT pathways for bone metabolism and their regulation by implant topography. European Cells & Materials, 24, 46–59. 10.22203/ecm.v024a04 22791372

[acel14139-bib-0012] Gaur, T. , Lengner, C. J. , Hovhannisyan, H. , Bhat, R. A. , Bodine, P. V. , Komm, B. S. , Javed, A. , van Wijnen, A. J. , Stein, J. L. , Stein, G. S. , & Lian, J. B. (2005). Canonical WNT signaling promotes osteogenesis by directly stimulating Runx2 gene expression. The Journal of Biological Chemistry, 280(39), 33132–33140. 10.1074/jbc.M500608200 16043491

[acel14139-bib-0013] Grillari, J. , Makitie, R. E. , Kocijan, R. , Haschka, J. , Vazquez, D. C. , Semmelrock, E. , & Hackl, M. (2021). Circulating miRNAs in bone health and disease. Bone, 145, 115787. 10.1016/j.bone.2020.115787 33301964

[acel14139-bib-0014] Grunewald, M. , Kumar, S. , Sharife, H. , Volinsky, E. , Gileles‐Hillel, A. , Licht, T. , Permyakova, A. , Hinden, L. , Azar, S. , Friedmann, Y. , Kupetz, P. , Tzuberi, R. , Anisimov, A. , Alitalo, K. , Horwitz, M. , Leebhoff, S. , Khoma, O. Z. , Hlushchuk, R. , Djonov, V. , … Keshet, E. (2021). Counteracting age‐related VEGF signaling insufficiency promotes healthy aging and extends life span. Science, 373(6554), 533–542. 10.1126/science.abc8479 34326210

[acel14139-bib-0015] Hamczyk, M. R. , Villa‐Bellosta, R. , Gonzalo, P. , Andres‐Manzano, M. J. , Nogales, P. , Bentzon, J. F. , Lopez‐Otin, C. , & Andres, V. (2018). Vascular smooth muscle‐specific Progerin expression accelerates atherosclerosis and death in a mouse model of Hutchinson‐Gilford progeria syndrome. Circulation, 138(3), 266–282. 10.1161/CIRCULATIONAHA.117.030856 29490993 PMC6075893

[acel14139-bib-0016] He, N. , Zhang, L. , Cui, J. , & Li, Z. (2014). Bone marrow vascular niche: Home for hematopoietic stem cells. Bone Marrow Research, 2014, 128436. 10.1155/2014/128436 24822129 PMC4009113

[acel14139-bib-0017] Heilmeier, U. , Hackl, M. , Schroeder, F. , Torabi, S. , Kapoor, P. , Vierlinger, K. , Eiriksdottir, G. , Gudmundsson, E. F. , Harris, T. B. , Gudnason, V. , Link, T. M. , Grillari, J. , & Schwartz, A. V. (2022). Circulating serum microRNAs including senescent miR‐31‐5p are associated with incident fragility fractures in older postmenopausal women with type 2 diabetes mellitus. Bone, 158, 116308. 10.1016/j.bone.2021.116308 35066213

[acel14139-bib-0018] Hemmatian, H. , Bakker, A. D. , Klein‐Nulend, J. , & van Lenthe, G. H. (2017). Aging, osteocytes, and Mechanotransduction. Current Osteoporosis Reports, 15(5), 401–411. 10.1007/s11914-017-0402-z 28891009 PMC5599455

[acel14139-bib-0019] Hernandez, L. , Roux, K. J. , Wong, E. S. M. , Mounkes, L. C. , Mutalif, R. , Navasankari, R. , Rai, B. , Cool, S. , Jeong, J. W. , Wang, H. , Lee, H. S. , Kozlov, S. , Grunert, M. , Keeble, T. , Jones, C. M. , Meta, M. D. , Young, S. G. , Daar, I. O. , Burke, B. , … Stewart, C. L. (2010). Functional coupling between the extracellular matrix and nuclear lamina by Wnt signaling in progeria. Developmental Cell, 19, 413–425. 10.1016/J.DEVCEL.2010.08.013 20833363 PMC2953243

[acel14139-bib-0020] Ho, Y. H. , del Toro, R. , Rivera‐Torres, J. , Rak, J. , Korn, C. , García‐García, A. , Macías, D. , González‐Gómez, C. , del Monte, A. , Wittner, M. , Waller, A. K. , Foster, H. R. , López‐Otín, C. , Johnson, R. S. , Nerlov, C. , Ghevaert, C. , Vainchenker, W. , Louache, F. , Andrés, V. , & Méndez‐Ferrer, S. (2019). Remodeling of bone marrow hematopoietic stem cell niches promotes myeloid cell expansion during premature or physiological aging. Cell Stem Cell, 25, 407–418.e6. 10.1016/J.STEM.2019.06.007 31303548 PMC6739444

[acel14139-bib-0021] Hofmann, J. W. , McBryan, T. , Adams, P. D. , & Sedivy, J. M. (2014). The effects of aging on the expression of Wnt pathway genes in mouse tissues. Age, 36(3), 9618. 10.1007/s11357-014-9618-3 24488586 PMC4082588

[acel14139-bib-0022] Kusumbe, A. P. , Ramasamy, S. K. , & Adams, R. H. (2014). Coupling of angiogenesis and osteogenesis by a specific vessel subtype in bone. Nature, 507, 323–328. 10.1038/nature13145 24646994 PMC4943525

[acel14139-bib-0023] Lehmann, J. , Narcisi, R. , Franceschini, N. , Chatzivasileiou, D. , Boer, C. G. , Koevoet, W. , Putavet, D. , Drabek, D. , van Haperen, R. , de Keizer, P. L. J. , van Osch, G. , & Ten Berge, D. (2022). WNT/beta‐catenin signalling interrupts a senescence‐induction cascade in human mesenchymal stem cells that restricts their expansion. Cellular and Molecular Life Sciences, 79(2), 82. 10.1007/s00018-021-04035-x 35048158 PMC8770385

[acel14139-bib-0024] Lewis, B. P. , Burge, C. B. , & Bartel, D. P. (2005). Conserved seed pairing, often flanked by adenosines, indicates that thousands of human genes are microRNA targets. Cell, 120(1), 15–20. 10.1016/j.cell.2004.12.035 15652477

[acel14139-bib-0025] Lopez‐Otin, C. , Blasco, M. A. , Partridge, L. , Serrano, M. , & Kroemer, G. (2023). Hallmarks of aging: An expanding universe. Cell, 186(2), 243–278. 10.1016/j.cell.2022.11.001 36599349

[acel14139-bib-0026] Manakanatas, C. , Ghadge, S. K. , Agic, A. , Sarigol, F. , Fichtinger, P. , Fischer, I. , Foisner, R. , & Osmanagic‐Myers, S. (2022). Endothelial and systemic upregulation of miR‐34a‐5p fine‐tunes senescence in progeria. Aging, 14, 195–224. 10.18632/aging.203820 35020601 PMC8791216

[acel14139-bib-0027] Matsuzaki, Y. , Mabuchi, Y. , & Okano, H. (2014). Leptin receptor makes its mark on MSCs. Cell Stem Cell, 15, 112–114. 10.1016/J.STEM.2014.07.001 25105573

[acel14139-bib-0028] Merideth, M. A. , Gordon, L. B. , Clauss, S. , Sachdev, V. , Smith, A. C. , Perry, M. B. , Brewer, C. C. , Zalewski, C. , Kim, H. J. , Solomon, B. , Brooks, B. P. , Gerber, L. H. , Turner, M. L. , Domingo, D. L. , Hart, T. C. , Graf, J. , Reynolds, J. C. , Gropman, A. , Yanovski, J. A. , … Introne, W. J. (2008). Phenotype and course of Hutchinson‐Gilford progeria syndrome. The New England Journal of Medicine, 358(6), 592–604. 10.1056/NEJMoa0706898 18256394 PMC2940940

[acel14139-bib-0029] Meshorer, E. , & Gruenbaum, Y. (2008). Gone with the Wnt/Notch: Stem cells in laminopathies, progeria, and aging. The Journal of Cell Biology, 181(1), 9–13. 10.1083/jcb.200802155 18378774 PMC2287275

[acel14139-bib-0030] Mo, L. , Ma, C. , Wang, Z. , Li, J. , He, W. , Niu, W. , Chen, Z. , Zhou, C. , & Liu, Y. (2022). Integrated Bioinformatic analysis of the shared molecular mechanisms between osteoporosis and atherosclerosis. Frontiers in Endocrinolog, 13, 950030. 10.3389/fendo.2022.950030 PMC9353191 35937806

[acel14139-bib-0031] Mojiri, A. , Walther, B. K. , Jiang, C. , Matrone, G. , Holgate, R. , Xu, Q. , Morales, E. , Wang, G. , Gu, J. , Wang, R. , & Cooke, J. P. (2021). Telomerase therapy reverses vascular senescence and extends lifespan in progeria mice. European Heart Journal, 42, 4352–4369. 10.1093/eurheartj/ehab547 34389865 PMC8603239

[acel14139-bib-0032] Osmanagic‐Myers, S. , Kiss, A. , Manakanatas, C. , Hamza, O. , Sedlmayer, F. , Szabo, P. L. , Fischer, I. , Fichtinger, P. , Podesser, B. K. , Eriksson, M. , & Foisner, R. (2019). Endothelial progerin expression causes cardiovascular pathology through an impaired mechanoresponse. The Journal of Clinical Investigation, 129(2), 531–545. 10.1172/JCI121297 30422822 PMC6355303

[acel14139-bib-0033] Primmer, S. R. , Liao, C. Y. , Kummert, O. M. P. , & Kennedy, B. K. (2022). Lamin A to Z in normal aging. Aging, 14(20), 8150–8166. 10.18632/aging.204342 36260869 PMC9648802

[acel14139-bib-0034] Sagelius, H. , Rosengardten, Y. , Hanif, M. , Erdos, M. R. , Rozell, B. , Collins, F. S. , & Eriksson, M. (2008). Targeted transgenic expression of the mutation causing Hutchinson‐Gilford progeria syndrome leads to proliferative and degenerative epidermal disease. Journal of Cell Science, 121(Pt 7), 969–978. 10.1242/jcs.022913 18334552

[acel14139-bib-0035] Schmidt, E. , Nilsson, O. , Koskela, A. , Tuukkanen, J. , Ohlsson, C. , Rozell, B. , & Eriksson, M. (2012). Expression of the Hutchinson‐Gilford progeria mutation during osteoblast development results in loss of osteocytes, irregular mineralization, and poor biomechanical properties. The Journal of Biological Chemistry, 287(40), 33512–33522. 10.1074/jbc.M112.366450 22893709 PMC3460452

[acel14139-bib-0036] Sun, J. F. , Phung, T. , Shiojima, I. , Felske, T. , Upalakalin, J. N. , Feng, D. , Kornaga, T. , Dor, T. , Dvorak, A. M. , Walsh, K. , & Benjamin, L. E. (2005). Microvascular patterning is controlled by fine‐tuning the Akt signal. Proceedings of the National Academy of Sciences of the United States of America, 102(1), 128–133. 10.1073/pnas.0403198102 15611473 PMC538747

[acel14139-bib-0037] Wang, Z. X. , Luo, Z. W. , Li, F. X. Z. , Cao, J. , Rao, S. S. , Liu, Y. W. , Wang, Y. Y. , Zhu, G. Q. , Gong, J. S. , Zou, J. T. , Wang, Q. , Tan, Y. J. , Zhang, Y. , Hu, Y. , Li, Y. Y. , Yin, H. , Wang, X. K. , He, Z. H. , Ren, L. , … Xie, H. (2022). Aged bone matrix‐derived extracellular vesicles as a messenger for calcification paradox. Nature Communications, 13, 1453. 10.1038/S41467-022-29191-X PMC8933454 35304471

[acel14139-bib-0038] Weigl, M. , Kocijan, R. , Ferguson, J. , Leinfellner, G. , Heimel, P. , Feichtinger, X. , Pietschmann, P. , Grillari, J. , Zwerina, J. , Redl, H. , & Hackl, M. (2021). Longitudinal changes of circulating miRNAs during bisphosphonate and Teriparatide treatment in an animal model of postmenopausal osteoporosis. Journal of Bone and Mineral Research, 36(6), 1131–1144. 10.1002/jbmr.4276 33598975 PMC8252367

[acel14139-bib-0039] Weilner, S. , Schraml, E. , Wieser, M. , Messner, P. , Schneider, K. , Wassermann, K. , Micutkova, L. , Fortschegger, K. , Maier, A. B. , Westendorp, R. , Resch, H. , Wolbank, S. , Redl, H. , Jansen‐Dürr, P. , Pietschmann, P. , Grillari‐Voglauer, R. , & Grillari, J. (2016). Secreted microvesicular miR‐31 inhibits osteogenic differentiation of mesenchymal stem cells. Aging Cell, 15, 744–754. 10.1111/ACEL.12484 27146333 PMC4933673

[acel14139-bib-0040] Whitmarsh, T. , Otake, Y. , Uemura, K. , Takao, M. , Sugano, N. , & Sato, Y. (2019). A cross‐sectional study on the age‐related cortical and trabecular bone changes at the femoral head in elderly female hip fracture patients. Scientific Reports, 9(1), 305. 10.1038/s41598-018-36299-y 30670734 PMC6343024

[acel14139-bib-0041] Wiley, C. D. , Flynn, J. M. , Morrissey, C. , Lebofsky, R. , Shuga, J. , Dong, X. , Unger, M. A. , Vijg, J. , Melov, S. , & Campisi, J. (2017). Analysis of individual cells identifies cell‐to‐cell variability following induction of cellular senescence. Aging Cell, 16(5), 1043–1050. 10.1111/acel.12632 28699239 PMC5595671

[acel14139-bib-0042] Xu, R. , Shen, X. , Si, Y. , Fu, Y. , Zhu, W. , Xiao, T. , Fu, Z. , Zhang, P. , Cheng, J. , & Jiang, H. (2018). MicroRNA‐31a‐5p from aging BMSCs links bone formation and resorption in the aged bone marrow microenvironment. Aging Cell, 17, e12794. 10.1111/ACEL.12794 29896785 PMC6052401

[acel14139-bib-0043] Zhang, L. , Pitcher, L. E. , Yousefzadeh, M. J. , Niedernhofer, L. J. , Robbins, P. D. , & Zhu, Y. (2022). Cellular senescence: A key therapeutic target in aging and diseases. The Journal of Clinical Investigation, 132(15), e158450. 10.1172/JCI158450 35912854 PMC9337830

[acel14139-bib-0044] Zhu, S. , Bennett, S. , Kuek, V. , Xiang, C. , Xu, H. , Rosen, V. , & Xu, J. (2020). Endothelial cells produce angiocrine factors to regulate bone and cartilage via versatile mechanisms. Theranostics, 10(13), 5957–5965. 10.7150/thno.45422 32483430 PMC7255007

