# Supplementary figures and images for: Aged‐vascular niche hinders osteogenesis of mesenchymal stem cells through paracrine repression of Wnt‐axis
Source: Aging Cell. 2024 Apr 5;23(6):e14139. doi: 10.1111/acel.14139 (PMC11166365; doi:10.1111/acel.14139)

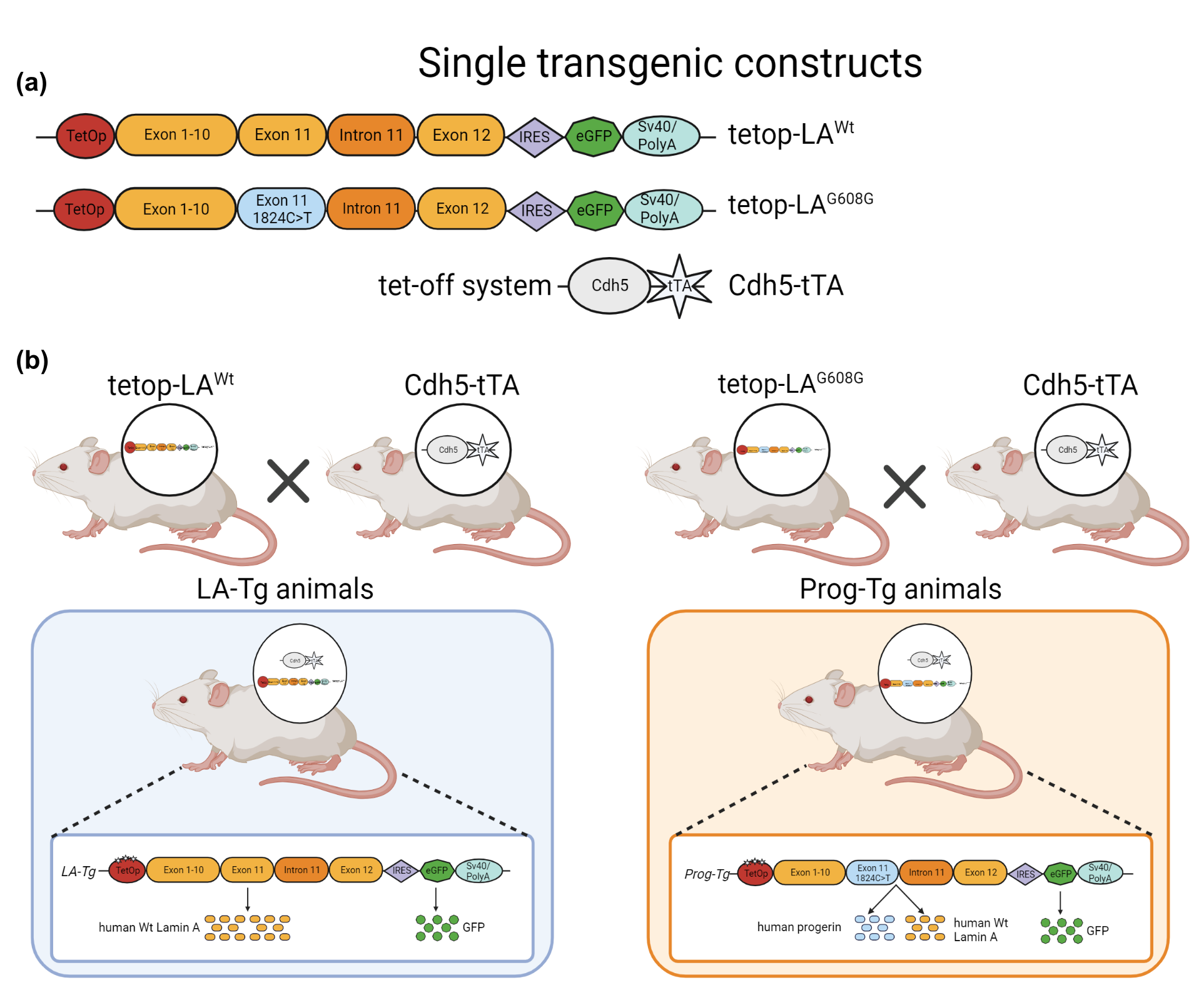

Supplement: Supplementary file 1 — Figure S1 [file ACEL-23-e14139-s004.tiff]

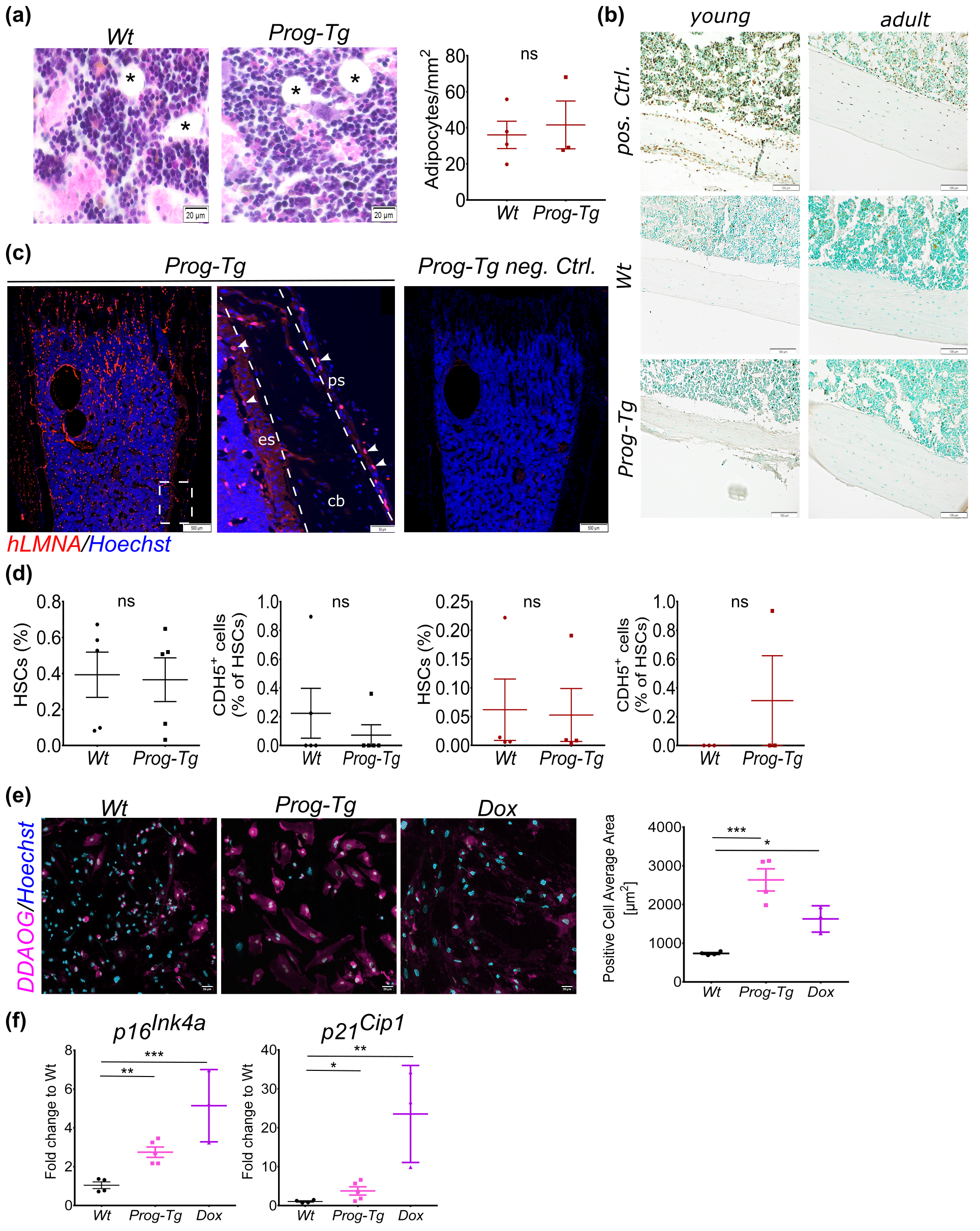

Supplement: Supplementary file 2 — Figure S2 [file ACEL-23-e14139-s001.tiff]

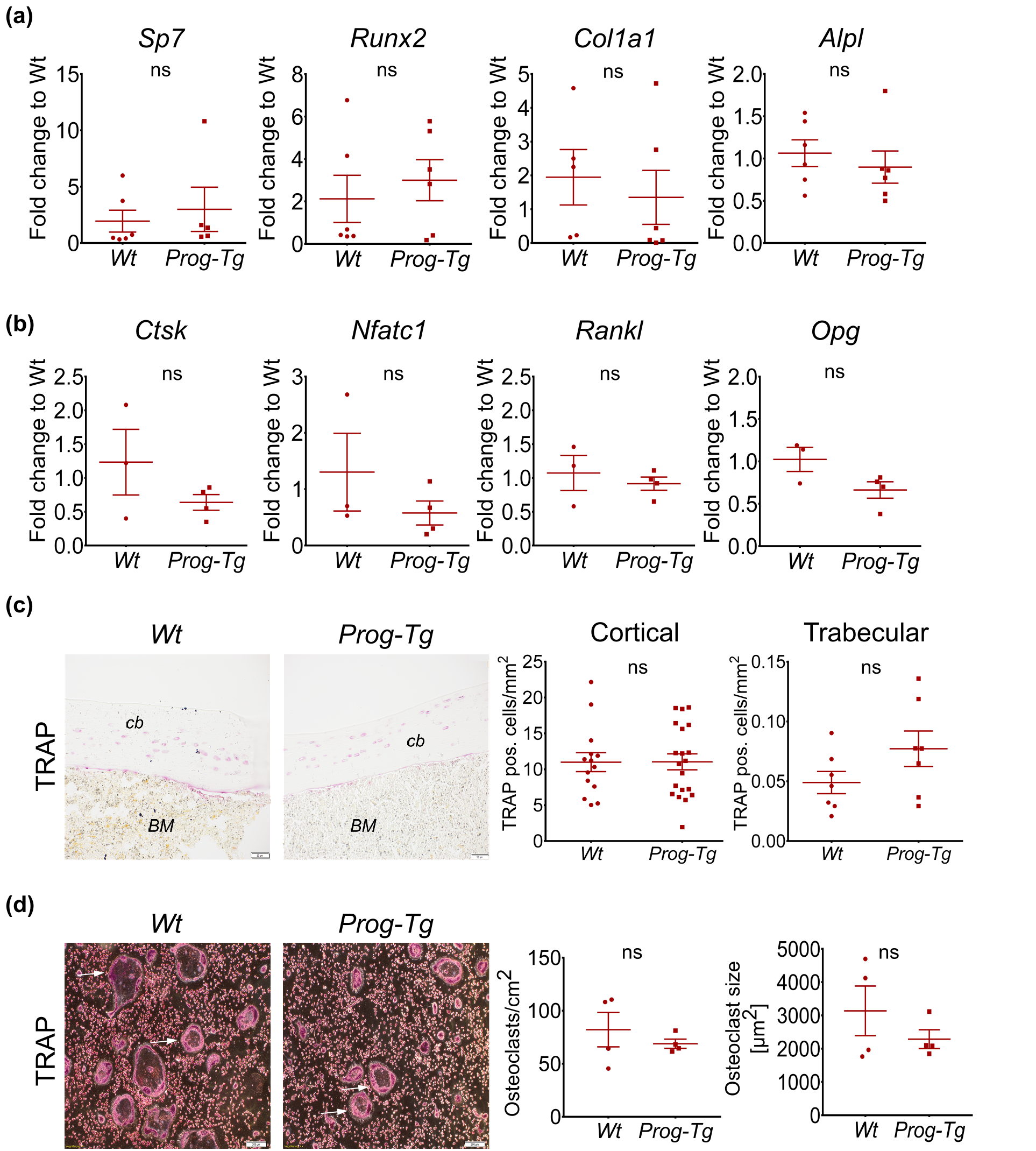

Supplement: Supplementary file 3 — Figure S3 [file ACEL-23-e14139-s003.tiff]

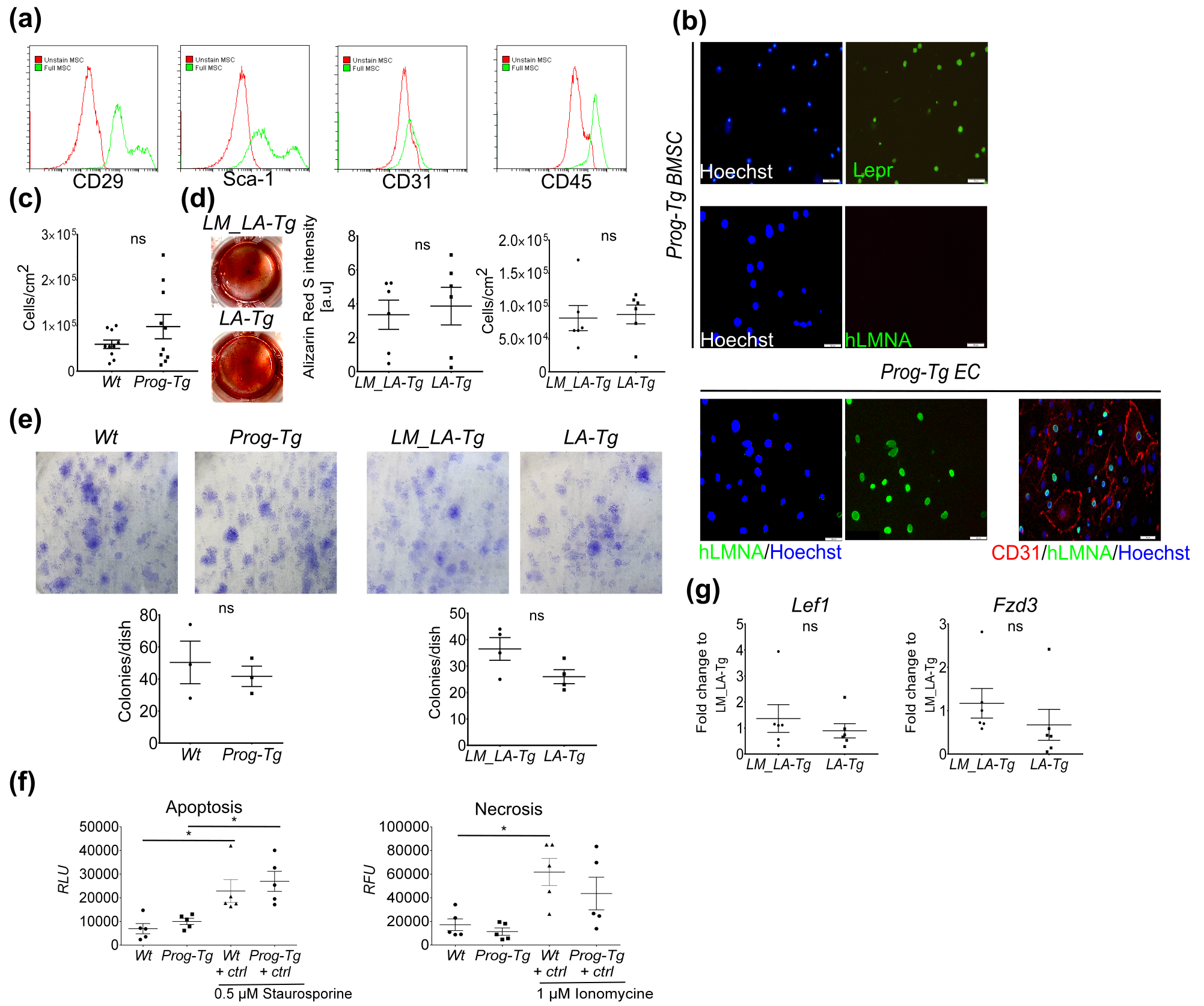

Supplement: Supplementary file 4 — Figure S4 [file ACEL-23-e14139-s006.tiff]

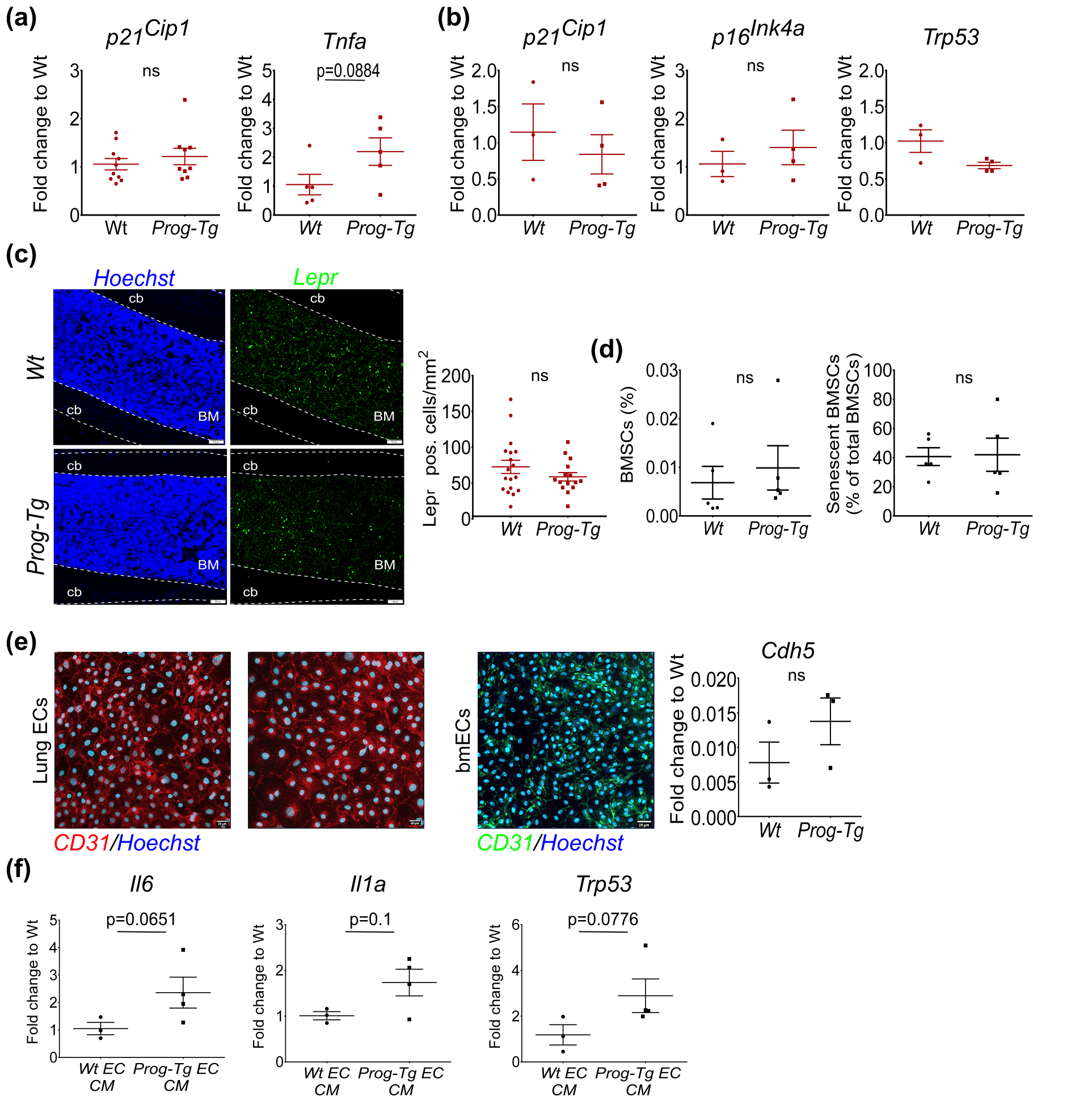

Supplement: Supplementary file 5 — Figure S5 [file ACEL-23-e14139-s002.tiff]

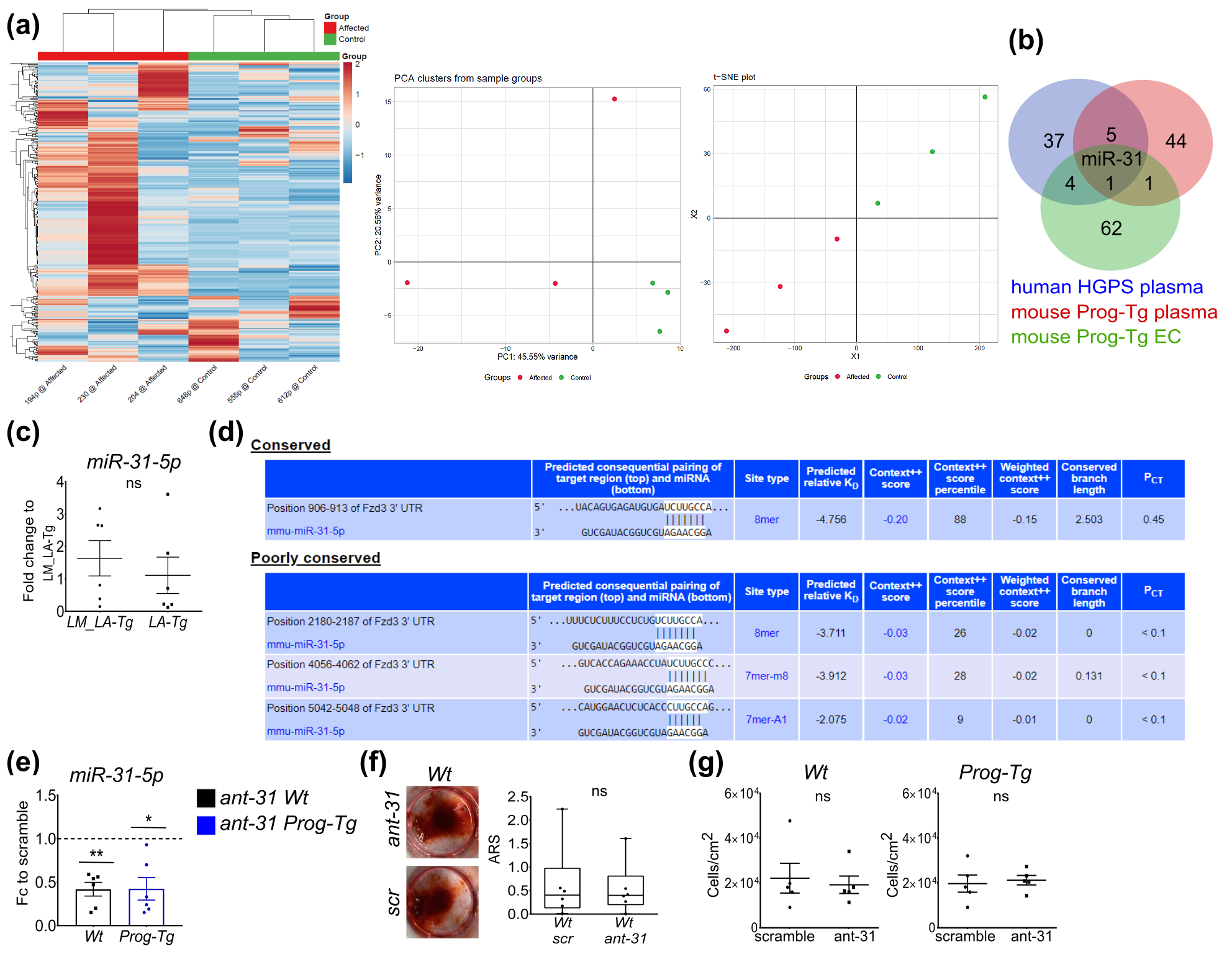

Supplement: Supplementary file 6 — Figure S6 [file ACEL-23-e14139-s007.tiff]
