# Supplementary material for: Aged‐vascular niche hinders osteogenesis of mesenchymal stem cells through paracrine repression of Wnt‐axis
Source: Aging Cell. 2024 Apr 5;23(6):e14139. doi: 10.1111/acel.14139 (PMC11166365; doi:10.1111/acel.14139)
Supplement: Supplementary file 7 — Table S1 [file ACEL-23-e14139-s005.docx]

**Supplementary Table 1. Trabecular bone parameters assessed by µCT measurements in tibia and lumbar vertebral body 4 (L4).**

| **Tibia** | | | | | | | |
| --- | --- | --- | --- | --- | --- | --- | --- |
|  | *Wt* | *Prog-Tg* | p value  *Wt* vs. *Prog-Tg* | *LM_LA-Tg* | *LA-Tg* | p value  *LM_LA-Tg* vs. *LA-Tg* | p value  *Prog-Tg* vs. *LA-Tg* |
| TV  [mm3] | 1,34 ± 0,47 | 1,40 ± 0,35 | ns | 1,75 ± 0,23 | 1,58 ± 0,50 | ns | ns |
| BV  [mm3] | 0,09 ± 0,04 | 0,10 ± 0,04 | ns | 0,19 ± 0,07 | 0,26 ± 0,14 | ns | ns |
| BV/TV | 0,08 ± 0,03 | 0,07 ± 0,02 | ns | 0,11 ± 0,03 | 0,15 ± 0,07 | ns | ns |
| Tb.N  [1/mm] | 1,93 ± 0,01 | 1,96 ± 0,77 | ns | 2,51 ± 0,60 | 3,35 ± 1,36 | ns | ns |
| Tb.Sp  [mm] | 0,04 ± 0,01 | 0,04 ± 0,01 | ns | 0,04 ± 0,01 | 0,05 ± 0,01 | ns | ns |
| Tb.Th  [mm] | 0,61 ± 0,30 | 0,59 ± 0,33 | ns | 0,40 ± 0,11 | 0,31 ± 0,15 | ns | ns |
| BMD  [mgHA/ccm] | 844 ± 55 | 847 ± 27 | ns | 852 ± 10 | 850 ± 11 | ns | ns |
| **L4 (4^th^ vertebral body)** | | | | | | | |
|  | *Wt* | *Prog-Tg* | p value  *Wt* vs. *Prog-Tg* | *LM_LA-Tg* | *LA-Tg* | p value  *LM_LA-Tg* vs. *LA-Tg* | p value  *Prog-Tg* vs. *LA-Tg* |
| TV  [mm3] | 2,79 ± 0,28 | 2,64 ± 0,33 | ns | 2,85 ± 0,24 | 2,71 ± 0,28 | ns | ns |
| BV  [mm3] | 0,44 ± 0,10 | 0,41 ± 0,11 | ns | 0,44 ± 0,05 | 0,62 ± 0,22 | ns | ns |
| BV/TV | 0,16 ± 0,04 | 0,16 ± 0,04 | ns | 0,16 ± 0,01 | 0,22 ± 0,06 | ns | ns |
| Tb.N  [1/mm] | 4,24 ± 0,96 | 4,26 ± 1,1 | ns | 3,89 ± 0,20 | 5,09 ± 0,96 | ns | ns |
| Tb.Sp  [mm] | 0,04 ± 0,01 | 0,04 ± 0,01 | ns | 0,04 ± 0,01 | 0,04 ± 0,01 | ns | ns |
| Tb.Th  [mm] | 0,21 ± 0,06 | 0,21 ± 0,06 | ns | 0,22 ± 0,01 | 0,16 ± 0,05 | ns | ns |
| BMD  [mgHA/ccm] | 861 ± 34 | 897 ± 35 | ns | 879 ± 8 | 898 ± 11 | ns | ns |

*Prog-Tg*, *LA-Tg* and corresponding *Wt* littermates (*LM*) were analyzed (age=35-40 weeks; n=8 *Prog-Tg,* n=9 *Wt* littermates (*Wt*), n=5 *LA‑Tg,* and n=5 for corresponding *Wt* littermate (*LM_LA-Tg*)). TV, total volume; BV, bone volume; BV/TV, bone volume fraction; Tb.N, trabecular number; Tb.Sp, trabecular spacing; Tb.Th, trabecular thickness; BMD, bone mineral density. Data presented as mean ± SD. Statistical analysis by one-way ANOVA followed by multiple comparisons post-hoc Tukey test (ns. not significant).

**Supplementary Table 2 (List of DE miRs in HGPS)**

| Human HGPS patient plasma | | | | |
| --- | --- | --- | --- | --- |
|  | **miRNA** | **logFC** | **P.Value** | **adj.P.Val** |
| 1 | hsa-miR-155-5p | 2,666655236 | 1.52857576656019e-9 | 3.06115593211956e-7 |
| 2 | hsa-miR-150-5p | 3,180185332 | 2.45875978483499e-9 | 3.06115593211956e-7 |
| 3 | hsa-miR-146b-5p | 1,774842505 | 4.69161887205674e-7 | 3,89404E-05 |
| 4 | hsa-let-7b-5p | -1,095095223 | 6,64395E-06 | 0,000413586 |
| 5 | hsa-miR-451a | -1,626976442 | 1,90898E-05 | 0,000950671 |
| 6 | hsa-miR-1275 | 4,691456033 | 7,15129E-05 | 0,002967785 |
| 7 | hsa-let-7c-5p | -1,766830743 | 0,000114145 | 0,004060315 |
| 8 | hsa-miR-98-5p | 1,705115068 | 0,000213844 | 0,006655908 |
| 9 | hsa-miR-574-3p | 11,4868459 | 0,00101718 | 0,025421394 |
| 10 | hsa-miR-181a-5p | 1,101781106 | 0,001113789 | 0,025421394 |
| 11 | hsa-miR-143-3p | -1,656073479 | 0,001123033 | 0,025421394 |
| 12 | hsa-miR-107 | -1,177396571 | 0,001621058 | 0,032877987 |
| 13 | hsa-miR-16-5p | -1,214497177 | 0,001805155 | 0,032877987 |
| 14 | hsa-miR-223-3p | 2,234108107 | 0,001848562 | 0,032877987 |
| 15 | hsa-miR-28-3p | 1,302297078 | 0,002279135 | 0,037833635 |
| 16 | hsa-miR-181b-5p | 1,449041649 | 0,002869786 | 0,044661052 |
| 17 | hsa-miR-361-3p | 3,101923584 | 0,003127523 | 0,04580902 |
| 18 | hsa-miR-30a-5p | -1,299498844 | 0,003464378 | 0,047923893 |
| 19 | hsa-miR-342-5p | 2,216299809 | 0,003765935 | 0,049211917 |
| 20 | hsa-miR-28-5p | 2,919435772 | 0,003952764 | 0,049211917 |
| 21 | hsa-miR-342-3p | 2,465246186 | 0,007487054 | 0,088775064 |
| 22 | hsa-miR-144-3p | -2,009842363 | 0,008110307 | 0,091793934 |
| 23 | hsa-miR-363-3p | -1,488493233 | 0,011419734 | 0,123631039 |
| 24 | hsa-miR-181c-3p | 10,64230672 | 0,013232144 | 0,135922549 |
| 25 | hsa-miR-92b-3p | -1,246767309 | 0,014099382 | 0,135922549 |
| 26 | hsa-miR-320b | -1,268988196 | 0,014192716 | 0,135922549 |
| 27 | hsa-miR-182-5p | -1,335670097 | 0,016617954 | 0,153254464 |
| 28 | hsa-miR-423-5p | -0,847418855 | 0,0174014 | 0,154748163 |
| 29 | hsa-miR-487b-3p | 10,44717206 | 0,019518934 | 0,167593606 |
| 30 | hsa-miR-103a-3p | -0,779991836 | 0,020198265 | 0,167645601 |
| 31 | hsa-miR-4286 | 10,38658439 | 0,021748578 | 0,174690193 |
| 32 | hsa-miR-93-5p | -0,919140396 | 0,026342898 | 0,202694247 |
| 33 | hsa-miR-144-5p | -1,380680231 | 0,026863093 | 0,202694247 |
| 34 | hsa-miR-486-3p | -1,294309472 | 0,030698921 | 0,21379621 |
| 35 | hsa-miR-183-5p | -1,354197797 | 0,032057748 | 0,21379621 |
| 36 | hsa-miR-423-3p | 1,038097391 | 0,032063084 | 0,21379621 |
| 37 | hsa-miR-7-1-3p | 10,13345088 | 0,032970196 | 0,21379621 |
| 38 | hsa-miR-4508 | -1,409521039 | 0,033358405 | 0,21379621 |
| 39 | hsa-miR-185-5p | -1,800163242 | 0,033486153 | 0,21379621 |
| 40 | hsa-miR-146a-5p | 0,832477611 | 0,036356885 | 0,219978381 |
| 41 | hsa-miR-889-3p | 10,05188404 | 0,036909233 | 0,219978381 |
| 42 | hsa-miR-21-3p | 10,03874157 | 0,037104787 | 0,219978381 |
|  | **miRNA** | **logFC** | **P.Value** | **adj.P.Val** |
| 43 | hsa-miR-101-3p | -0,944633022 | 0,040089756 | 0,232147659 |
| 44 | hsa-miR-31-5p | 9,895529525 | 0,044573152 | 0,252243517 |
| 45 | hsa-miR-766-3p | 6,379366437 | 0,045592407 | 0,252277984 |
| 46 | hsa-miR-181a-2-3p | 1,651165437 | 0,049521703 | 0,263252981 |
| 47 | hsa-miR-382-3p | 3,916862637 | 0,049690322 | 0,263252981 |

**Supplementary Table 3. Primers used for quantitative real-time PCR analysis.**

| Gene | Genbank accession number | Primer Sequences |
| --- | --- | --- |
| *Hprt* | NM_013556.2 | Forward: 5’-GCAGTCCCAGCGTCGTGATTA-3’  Reverse: 5’-TGATGGCCTCCCATCTCCTTCA-3’ |
| *Runx2* | NM_001146038.2 | Forward: 5’-CGAAATGCCTCCGCTGTTAT-3’  Reverse: 5’-TGTCTGTGCCTTCTTGGTTCC-3’ |
| *Sp7* | NM_130458.4 | Forward: 5’-GTCCTCTCTGCTTGAGGAAGAA-3’  Reverse: 5’-GGGCTGAAAGGTCAGCGTAT-3’ |
| *Col1a1* | NM_007742.4 | Forward: 5’-GGTCCACAAGGTTTCCAAGG-3’  Reverse: 5’-GTTCCAGGCAATCCACGAG-3’ |
| *Alpl* | NM_007431.3 | Forward: 5’-CCTGACTGACCCTTCGCTCT-3’  Reverse: 5’-CCATCTCCACTGCTTCATGC-3’ |
| *Ocn* | NM_007541.3 | Forward: 5’-GCAGGAGGGCAATAAGGTAG-3’  Reverse: 5’-CTTTAGGGCAGCACAGGTC-3’ |
| *Dmp1* | NM_001359013.1 | Forward: 5’-GTTCCTTTGGGGGCTGTC-3’  Reverse: 5’-CTATTTGCCTGTCCCTCTGG-3’ |
| *Mepe* | NM_053172.2 | Forward: 5’- TGTTGGACTGCTCCTCTTCA-3’  Reverse: 5’- CCATCCTCTGTGCCTTCATC-3’ |
| *Sost* | NM_024449.6 | Forward: 5’-TTCAGGAATGATGCCACAGA-3’  Reverse: 5’-GTCAGGAAGCGGGTGTAGTG-3’ |
| *p16^Ink4a^* | NM_009877.2 | Forward: 5’-AGAGCGGGGACATCAAGAC-3’  Reverse: 5’-CTGAGGCCGGATTTAGCTC-3’ |
| *p21^Cip1^* | NM_007669.5 | Forward: 5’-TGCCAGCAGAATAAAAGGTG-3’  Reverse: 5’-TTGCTCCTGTGCGGAAC-3’ |
| *Trp53* | NM_011640.3 | Forward: 5’-ACAGCACATGACGGAGGTC-3’  Reverse: 5’-CTCGGGTGGCTCATAAGGTA-3’ |
| *Il1a* | NM_010554.4 | Forward: 5’-TGCCATTGACCATCTCTCTCT-3’  Reverse: 5’-GATACTGTCACCCGGCTCTC-3’ |
| *Il6* | NM_031168.2 | Forward: 5’-GGGAAATCGTGGAAATGAGA-3’  Reverse: 5’-TCCAGTTTGGTAGCATCCATC-3’ |
| *Tnfa* | NM_013693.3 | Forward: 5’-CCCCAAAGGGATGAGAAGTT-3’  Reverse: 5’-TGGGCTACAGGCTTGTCACT-3’ |
| *Lef1* | NM_010703.5 | Forward: 5’-AGCCTGTTTATCCCATCACG-3’  Reverse: 5’-GGGTGCTCCTGTTTGACCT-3’ |
| *Fzd3* | NM_021458.2 | Forward: 5’-GAAGCAAAGCAGGGAGTGTC-3’  Reverse: 5’-CTCCATTCCTCGGTAACTGC-3’ |
| *Ctsk* | NM_007802.4 | Forward: 5’-TAGCCACGCTTCCTATCCGA-3’  Reverse: 5’-CCGAGAGATTTCATCCACCTTG-3’ |
| *Nfatc1* | NM_016791.4 | Forward: 5’-CCCGGAGTTCGACTTCGATT-3’  Reverse: 5’-CATAACTGTAGTGTTCTGCGGC-3’ |
| *Tnfrsf11b* | NM_008764.4 | Forward: 5’-AGCTGCTGAAGCTGTGGAAA-3’  Reverse: 5’-CTGCTCTGTGGTGAGGTTCG-3’ |
| *Tnfsf11* | NM_011613.4 | Forward: 5’-TTGCACACCTCACCATCAAT-3’  Reverse: 5’-CCCTTAGTTTTCCGTTGCTT-3’ |
| *Il1b* | NM_008361.4 | Forward: 5’-GCCACCTTTTGACAGTGATGAG-3’  Reverse: 5’-TGATGTGCTGCTGCGAGATT-3’ |
| *Ccl20* | NM_016960.2 | Forward: 5’-TCCTTGCTTTGGCATGGGTA-3’  Reverse: 5’-TCTTAGGCTGAGGAGGTTCACA-3’ |
