# Supplementary material for: Aged‐vascular niche hinders osteogenesis of mesenchymal stem cells through paracrine repression of Wnt‐axis
Source: Aging Cell. 2024 Apr 5;23(6):e14139. doi: 10.1111/acel.14139 (PMC11166365; doi:10.1111/acel.14139)
Supplement: Supplementary file 8 — Appendix S1 [file ACEL-23-e14139-s008.docx]

**Supplementary material**

**Aged vascular niche hinders osteogenesis of mesenchymal stem cells through paracrine repression of Wnt-axis**

**Contents**

- Supplementary figure legends
- Supplementary figures 1-6
- Experimental procedures
- Supplementary Table 1 (Trabecular parameters of µCT measurements)
- Supplementary Table 2 (List of DE miRs in HGPS)
- Supplementary Table 3 (List of primers)
- References

# Supplementary figure legends

Figure S1. Scheme for lamin A minigene constructs used to generate *LA-Tg* and *Prog-Tg* animals. **(a)** Constructs with human wild-type lamin A and mutated lamin A *(*1824C>T *in* HGPS patients) minigenes, under the control of the promotor with tetracycline-responsive element *(tetop),* were used to generate single transgenic tetop-LA^Wt^ (referred *in text* as *LM_LA-Tg* controls) and single transgenic tetop-LA^G608G^ mice (referred as *Wt* controls), respectively. Constructs generated by Sagelius et al., (Sagelius et al., 2008) contain exons 1-10, exon 11, intron 11, and exon 12 of the human lamin A gene followed by internal ribosomal entry site (IRES), a coding region for eGFP and a Sv40 poly A site. Tetop-LA-G608G construct carries in *the* exon 11 the most common HGPS mutation (1824C>T). Note that in the absence of tetracycline-responsive transcriptional activator (tTA) no expression of *Wt* and mutated lamin A minigenes is detected in respective single transgenic control animals as described in (Osmanagic-Myers et al., 2019; Sagelius et al., 2008). **(b)** Single transgenic mice tetop-LA^Wt^ or tetop-LA^G608G^ were crossed with endothelial transactivator mice Cdh5-*tTA* harboring tTA under the control of the endothelial-specific cadherin 5 (Cdh5) promoter (see (Gory et al., 1999; Sun et al., 2005) to generate bi-transgenic *LA-Tg* and *Prog-Tg* mice, respectively. *LA-Tg* mice express specifically in the endothelium only *Wt* human lamin A whereas *Prog-Tg* mice express mutated form of the human lamin A (progerin) as well as *Wt* lamin A. Note, that in the presence of doxycycline, the transcription of the lamin A minigene is switched off.

Figure S2. Evaluation of bone marrow adiposity, apoptosis and senescence DDAOG staining. **(a)** HE-stained tibiae representative sections used to assess bone marrow adiposity with marked adipocytes (asterisk) in *Wt* and *Prog-Tg* animals. Right panel, mean number of adipocytes per animal measured per mm^2^ area (age=30-35 weeks, n=3-4). Scale bar, 20 µm. **(b)** Representative images of TUNEL assay on whole bone sections of young and adult *Wt* and *Prog-Tg* animals (age ≤ 14 days for young, age=30-35 weeks for adult). **(c)** Representative immunofluorescence images of whole bone sections in young *Prog-Tg* mice using anti-human lamin A (hLMNA) staining and Hoechst with corresponding negative controls. Arrowheads in magnified regions point to hLMNA-stained nuclei (progerin) aligning in the vasculature at periosteum (ps) and at endosteum (es) as well. cb, cortical bone **(d)** Immunophenotypic analysis of hematopoietic stem cells in the whole bone marrow compartment by flow cytometry. Quantification of Lin^-^c-Kit^+^Sca-1^+^ HSC subpopulation and CDH5 expressing HSCs as percentage of total HSC population derived from young and adult animals (age ≤ 14 days (black), n=5*,* age=30-35 weeks (red), n=4 per genotype). **(e)** Representative images of DDAOG staining in lung ECs derived from *Wt,* *Prog-Tg* animals and *Wt* ECs treated with Doxorubicin to induce senescence. Right panel, quantification of DDAOG^+^ cell areas (age ≤ 14 days, n=3-5). One-way ANOVA with posthoc Tukey´s multiple comparisons test. **(f)** Gene expression levels of senescence markers in lung ECs extracts and *Wt* ECs treated with Doxorubicin (age ≤ 14 days, n=3-5). Statistical analysis by unpaired two-tailed Student´s *t*-test (*p<0.05, **p<0.01, ***p<0.001, ns. not significant).

# Figure S3. Evaluation of osteogenesis genes in bones and bone marrow extracts and osteoclastogenesis of HSCs. Gene expression levels of (a) osteogenic markers in the bone marrow and (b) osteoclast genes in long bones of adult *Wt* and *Prog-Tg* animals, respectively (age=30-35 weeks; n=3-6). (c) Representative images of TRAP staining in cortical bone of adult animals. Right panel shows quantification of TRAP^+^ cells in cortical and trabecular bone sections. Each dot represents results from a region of interest (ROI). 4-8 and 2-3 ROIs were analyzed per biological replicate for cortical and trabecular bone with total ROI ≥14 and 7 per genotype, respectively (age=30-35 weeks, n=3-4 animals). BM, bone marrow; cb, cortical bone. Scale bar, 50 μm. (d) Representative images of TRAP-stained osteoclasts differentiated from HSCs derived from *Wt* and *Prog-Tg* animals. Right panel shows quantification of osteoclast number and size (age=30-35 weeks, n=4). Scale bar, 200 μm. Statistical analysis using unpaired two-tailed Student´s *t*-test (ns, not significant).

Figure S4. Characterization of enriched BMSCs. (a) Phenotypic flow cytometry characterization of enriched adherent BMSCs four days after isolation (age ≤14 days). **(b)** Representative immunofluorescence images showing positive staining for MSC lineage marker Lepr in BMSCs after enrichment. Lower panel, absence of human lamin A (hLMNA) expression in enriched BMSCs in contrast to evident staining in ECs derived from *Prog-Tg* mice. Scale bar, 100 µm. Note that endothelial specificity is confirmed using CD31 positive staining of cell junctions. Scale bars, 50 μm and 100 µm. **(c)** BMSCs derived either from *Prog-Tg* mice and their respective *Wt* littermates were subjected to osteoblast differentiation followed by quantification of cell numbers on day 14 of osteogenic differentiation (age ≤14 day, n=10). **(d)** Representative images of ARS stained deposited mineralized matrix and quantification after 14 days of osteoblast differentiation of BMSCs isolated from *LA-Tg* control animals and corresponding *LM_LA-Tg* littermates (age ≤14 days, n=6) and quantification of cell numbers on day 14. **(e)** Representative images of crystal violet stained cell colonies derived from BMSCs subjected to colony forming unit assay. Lower panel, quantification of colony forming units of BMSCs (age ≤14 days, n=3; n=4 *LA-Tg* and their *LM_LA-Tg* littermates). **(f)** Assessment of apoptosis and necrosis using annexin V-complementary NanoBIT luciferase assay with profluorescent DNA dye (n=5). Relative luminescence units (RLU) and relative fluorescence units (RFU) are shown on the Y-axis. One-way ANOVA with posthoc Tukey´s multiple comparisons test. **(g)** Gene expression levels of *Lef1 and Fzd3* in BMSCs isolated from *LA-Tg* animals and their corresponding littermates *LM_LA-Tg* (age≤14 days, n=6). Statistical analysis by unpaired two-tailed Student´s *t*-test. ns, not significant.

Figure S5. Paracrine effects of senescent vascular niche (a, b) Gene expression levels of senescence markers in **(a)** EC-depleted bone marrow and **(b)** in BM-depleted bone extracts (age=30-35 weeks, n=3-10). **(c)** Lepr positive BMSC populations in whole bone sections of adult mice detected using immunofluorescence microscopy. Right panel, quantification of Lepr^+^ cells (age=30-35 weeks; n=3, ROI ≥15 per genotype. Scale bar, 100 µm). **(d)** Flow cytometry analysis of CD45-Sca-1+PDGFRα+ BMSCs and senescent CD45^-^Sca-1^+^PDGFRα^+^DDAOG^+^ BMSCs (as proportion of total CD45^-^Sca-1^+^PDGFRα^+^ BMSCs) in the BM of young *Wt* and *Prog-Tg* animals analyzed by DDAOG far-red probe (age≤14 days, n=5). **(e)** Purity assessment of ECs derived from lung and BM using endothelial specific CD31 staining of cell-cell junctions and for BM-ECs additionally by gene expression analysis of Cdh5 levels (age≤14 days, n=3). **(f)** Gene expression levels of SASP markers *Il6, Il1a* and *Trp53* after osteogenic differentiation of young *Wt* BMSCs (age≤14 days) in the presence of conditioned media collected from senescent (*Prog-Tg* EC CM) and non-senescent (*Wt* EC CM), respectively (n=3-4). Statistical analysis by unpaired two-tailed Student´s *t*-test (ns, not significant).

**Figure S6. miR profiling in HGPS plasma and miR-31-5p target analysis.** **(a)** Heatmap showing clustering of differentially expressed miRs in HGPS patients (n=3, Red) and age-and gender matched controls (n=3, Green). Data is based on RPM normalized reads and scaled using the unit variance method for visualization in heatmaps. Clustering is done using the average method of heatmap, calculating the distances as correlations. Principal component analysis (PC) and t-SNE analysis are shown to the right. **(b)** VENN diagram exhibiting overlap of differentially expressed miRs in plasma from HGPS patients, mouse plasma and ECs isolated from *Prog-Tg* animals. **(c)** Gene expression levels of miR-31-5p in BMSCs isolated from *LA-Tg* animals and their corresponding littermates *LM_LA-Tg* (age≤14 days, n=6). **(d)** Predicted binding sites of miR-31-5p in 3´UTR of *Fzd3* (TargetScanMouse version 8.0). **(e)** Reduced gene expression levels of miR-31-5p in BMSCs derived from *Wt* and *Prog-Tg* 48h after antimiR-31 (ant-31) treatment shown as fold change to scramble (age ≤14 days, n=6). Paired Student´s *t*-test (anti-miR-31-5p treated vs. scramble) (*p<0.05, **p<0.01). **(f)** Osteogenic differentiation of scramble- or anti miR 31 treated BMSCs (ant-31) derived from *Wt* animals (age ≤14 days, n=6). Wilcoxon Signed Rank test (anti-miR-31-5p treated vs. scramble). **(g)** Analysis of cell numbers in *Wt* and *Prog-Tg* BMSCs, after scramble or anti-mir-31 treatment, on day 14 of osteogenic differentiation (age ≤14 days, n=5). **(c,g)** Unpaired two tailed Student´s *t*-test (ns. not significant).

# EXPERIMENTAL PROCEDURES

# Animals

Bi-transgenic *Prog-Tg* and *LA-Tg* mice were generated by crossing tet-operon-driven transgenic mice (C57BL/6J background) harboring the wild-type (tetop-LA^Wt^) or HGPS mutant (1824C>T; G608G; tetop-LAG608G) lamin A minigene with transgenic mice expressing a tetracycline-responsive (TA responsive) transcriptional activator under the control of the EC specific VE-cadherin promoter (Cdh5-tTA mice, Jackson Laboratories MGI:4437711, FVB background) as described (Manakanatas et al., 2022; Osmanagic-Myers et al., 2019) and explained in detail (Figure S1). Bi-transgenic *Prog-Tg* and *LA-Tg* animals were maintained at C57BL/6J background (N5 generation; ~97%). *LA-Tg* and *Prog-Tg* mice were kept in the absence of doxycycline to allow for constitutive expression of Wt or mutated lamin A minigenes. Young animals were considered at the age between 10 and 14 days (≤14 days), whereas adult animals were considered at the age ≥ 20 weeks with the exact age information provided in the corresponding figure legend. For physiological aging experiment young *Wt* animals were used at the age of ~3 months, whereas aged were used at ~22 months (C57BL/6J background). Mixed genders with similar proportion of females and males were used for all experiments.

# Endothelial cell isolation

Primary endothelial cells (ECs) were isolated and cultured as described previously (Osmanagic-Myers et al., 2019). Briefly, lungs from young animals (age ≤14 days) were collected and treated with 200 U/ml collagenase I (Gibco, 17100-017) for 45 minutes at 37°C, passed through a 19-gauge needle and filtered through a 70 µm cell strainer to obtain single cell suspensions. After centrifugation, cells were seeded on gelatin-coated (1% w/v) and fibronectin-coated (1 µg/ml) plates and cultured in DMEM supplemented with 20% fetal calf serum (FCS), EC growth supplement (CellBiologics, CatNo:1166), 25 mM HEPES, 50 U/ml penicillin, 50 μg/ml streptomycin, 2 mM L-glutamine, 1 mM nonessential amino acids,1 mM sodium pyruvate, and 139 μg/ml heparin (complete culture medium). After two days in culture, ECs were isolated using magnetic bead sorting with rat anti-ICAM2 antibodies (BD Biosciences, clone 3C4 [mlC2/4], 553326) coupled to sheep anti-rat IgG paramagnetic Dyna-beads (Invitrogen, 11035). ECs were kept at mild hypoxic conditions (10% oxygen) for amplification and transferred to normoxia when they reached confluence.

# Bone homogenate preparation and bone marrow EC isolation

Bone tissue was separated from bone marrow and homogenized using ceramic beads (Bertin Instruments, P000911-LYSK0-A) in Qiazol (Qiagen RNeasy Kit) in a Precellys 24 tissue homogenizer according to manufacturer´s protocol. For gene expression analysis, bone marrow (BM) was depleted of bone marrow ECs (bmECs) and bmECs cultured according to adopted protocols (Poulos et al., 2017; Smith et al., 2021). Briefly, magnetic bead sorting with rat anti-mouse CD31 antibodies (BD Biosciences, 557355) coupled to sheep anti-rat IgG paramagnetic Dynabeads (Invitrogen, 11035) was used to obtain supernatants defined as BM depleted of bmECs. For culture separated bmECs were seeded on gelatin (1% v/w) and fibronectin (20 µg/mL) coated 48-well plates. In order to sufficiently amplify for collection of conditioned media, bmECs were kept in mild hypoxia (~10% oxygen) for the total duration of 2-3 weeks.

# Collection of conditioned media for assessment of paracrine effects on BMSCs

Conditioned media (CM) were prepared as previously described (Manakanatas et al., 2022). Briefly, prior addition to primary EC cultures, complete culture media (see above) were first depleted of microRNAs using centrifugation at 100.000 x g for 24 h at 4°C. ECs were seeded at equal densities and cultured in these miR-depleted CMs, allowing secretion for 48 h. Every second day, CMs were collected and centrifuged at 5000 x g to remove all aggregates and cell debris with subsequent filtering through 0.22 µm filter. Conditioned media were collected every two days for the duration of 2-3 weeks and all fractions were pooled after completion of the bmEC culture and normalized to total cell counts. EC used for collection of CMs were subsequently checked for purity by immunofluorescence microscopy using endothelial PECAM-1 (CD31) specific antibodies (see below) or preparation of cell extract for RNA isolation and gene expression analysis.

Subsequently, CMs were mixed in the ratio 1:2 with the osteogenic differentiation media and added to freshly isolated adherent BMSC cultures (see below). During the 14-day osteogenic differentiation, CM-osteogenic media were replenished every three days.

# BMSC isolation and culture

Bone marrow mesenchymal stem cells (BMSCs) were isolated from the bone marrow (BM) of long bones of young *Wt* and *Prog-Tg* animals using slightly adopted protocols as previously described (Wang et al., 2022). Briefly, BM was flushed with using a 21-gauge needle and filtered through a 70 µm cell strainer to obtain single cell suspensions. After centrifugation, cells were seeded at ~2.2*10^6^ cells/cm^2^ and left to grow for four days in DMEM supplemented with 10% FCS, 50 U/ml penicillin, and 50 μg/ml streptomycin. After removal of non-adherent cells, BMSC-enrichment was confirmed by BMSC specific-marker staining using mouse anti-leptin receptor antibody (R&D Systems, AF497-SP) by immunofluorescence microscopy and flow cytometry analysis of BMSC lineage marker.

# Analysis of bone marrow cell populations and in vivo detection of DDAOG-senescent cell fractions using flow cytometry

Cells were collected by flushing bone marrow of long bones using a 21-gauge needle, then filtering through a 70 µm cell strainer followed by centrifugation and addition of red blood cell lysis buffer (Invitrogen, #00-4300-54) for erythrocyte lysis according to manufacturer´s protocol. Detection of senescence-associated beta galactosidase (SA-β-gal) activity in vivo was adopted according to previously published reports (Debacq-Chainiaux et al., 2009; Flor et al., 2022). Briefly, freshly isolated single bone marrow suspensions, were incubated with 1 µM bafilomycin for 30 min to induce lysosomal alkalization, followed by one hour incubation either with vehicle only or with a far-red 9H-(1,3-dichloro-9,9-dimethylacridin-2-one; DDAO)-galactoside (DDAOG) probe at 37°C protected from light to stain senescent cells as described previously (Flor et al., 2022). Equal number of cells were incubated with fluorophore conjugated antibodies in PBS with 1% bovine serum albumin (BSA) for 30 minutes at 4°C (see below). Thereafter, cells were stained with Zombi violet NIR (423105) solution for 15 min at RT to distinguish live and dead cell populations. Samples were acquired on Cytoflex S flow cytometer using CytExpert software (Beckman Coulter). Data analysis was performed using the CytExpert software. For analysis of target populations, first doublet exclusion was performed with subsequent analysis on viable cell populations according to Zombi NIR staining. Total bone ECs were gated as CD45^-^Ter^-^119^-^CD31^+^ and shown as proportion of total CD45^-^ population in percent as described in (Kusumbe et al., 2014). Hematopoietic stem cell (HSC) panel was designed according to HSC specific expression of tyrosine kinase receptor c-Kit and the membrane glycoprotein Sca-1, lacking the markers of terminal differentiation CD3εLy-6/Ly-6C (Gr-1), CD11b, CD45R (B220), Ter-119 lineage markers (termed Lin^-^) and gating performed as previously reported (Chen et al., 2020; Rossi et al., 2011). Accordingly, HSC populations were gated as Lin^-^ Sca-1^+^c-Kit^+^ population. BMSCs populations were gated as CD45^-^PDGFRa^+^ Sca-1^+^ as described in (Houlihan et al., 2012). Senescent DDAOG^+^ populations were gated on far-red (670 nm) to obtain the percentage of respective senescent cell types as described in (Flor et al., 2022). The following antibodies were purchased from BioLegend except (rat anti-mouse Emcn (PE, SantaCruz Biotechnology, #sc-65495)): rat anti-mouse CD45 (AF700,#147716), rat anti-mouse Ter-119 (AF700, #116220), rat anti-mouse CD117 (c-kit, AF700, #105845), rat anti-mouse CD140a (PDGFRα, BV421, #135923), rat anti-mouse CD144 (Cdh5, BV421, #138013), Lin-negative (Lin^-^) panel (PE, #133303), rat anti-mouse Sca-1 (Ly-6A/E, PE/Dazzle, #108138), rat anti-mouse CD31 (PerCP/Cyanine 5.5, #102522).

# Osteogenic differentiation

Enriched BMSCs, four days after isolation from the BM (see above) were incubated in DMEM (Merck) with 10% FCS, 2 mM L-glutamine, 50 U/ml penicillin, 50 μg/ml streptomycin, 10 mM glycerophosphate and 50 µg ascorbic acid (osteogenic medium) to induce osteogenic differentiation as described previously (Hu et al., 2018). Briefly, osteogenic medium was changed twice per week during the whole osteogenic differentiation period that lasted 14 days. For detecting the mineralized matrix formation, cells were fixed with 4% paraformaldehyde (PFA) and stained on day 14, with 1% Alizarin Red S (ARS; Merck) for 30 min. For quantification, the staining was dissolved in a hydrochloric acid (0.1 M)/sodium dodecyl sulfate (0.5%) buffer followed by extinction measurements at 405 nm and normalized to viable cells determined by cell count at the end of the experiment. For gene expression analysis of osteogenic genes, cells were collected at day 14 and processed for RT-qPCR.

# Doxorubicin treatment

Endothelial cells isolated from lungs of wild-type mice were seeded at ~4.5*10^5^ cells/cm^2^ in 48-well plates in complete culture medium (see above) and treated with 0.1 µM doxorubicin (Sigma, D1515) for 2 hours on two sequential days. Five days following the last doxorubicin treatment, total RNA was isolated using the miRNeasy Mini Kit (Qiagen 217084) for gene expression analysis (see below).

# antimiR-31 transfection

Four days following cell attachment, isolated BMSCs (see above) were transfected for 48 h with scramble (control) (Dharmacon, CN-001000-01-05) or antimiR-31-5p (Dharmacon, IH 310524 07-0005) using Lipofectamine reagent (LipofectMax, ABP Biosciences, FP310) with subsequent 14-day osteogenic differentiation. Thereafter, ARS-stained mineralized matrix depositions and gene expression of osteogenic markers were analyzed (see above).

# Immunofluorescence microscopy

Cultured ECs derived from lung or bone marrow or BMSCs were washed twice with PBS following fixation in 4% paraformaldehyde (PFA) for 10 min, incubation in 0.1 M glycine in PBS for 10 min, permeabilization in 0.5% Triton X-100 in PBS for 5 min and blocking in 3% BSA in PBS for 1 h. Following blocking, cells were then incubated with primary antibodies diluted in blocking buffer with 3% BSA, 0.1% Tween20 in PBS at 4°C overnight. After washing, samples were incubated for 1 h with fluorescently labeled secondary antibodies followed by washing with PBS. For DNA staining, cells were incubated with Hoechst (1:10000 in PBS) for 10 minutes at room temperature (RT) and mounted in ibidi mounting medium (Ibidi, 50001). Images were acquired on Olympus BX63 imaging system or subjected to high throughput image analysis using ImageXpress Pico system (see below). The following primary antibodies

were used: mouse monoclonal antibodies: mouse monoclonal anti-human Lamin A/C (JOL2) (1:50; clone JoL2, mab3211, abcam), mouse anti-mouse beta catenin antibody (Santa Cruz, sc-7963) and goat monoclonal anti-mouse Lef1 (1:30; Thermo Fisher Scientific, MA1-12432). Following secondary antibodies were used: goat anti-mouse Alexa 594, donkey anti-goat Alexa 488 (DyLight Fluor secondary antibodies, Thermo Fisher Scientific).

# CHIR99021 treatment

For assessment of Wnt activity on the osteogenic differentiation potential, enriched BMSCs were cultured in osteogenic differentiation medium containing selective GSK-3b inhibitor CHIR99021 (2,5 µM) or DMSO (untreated) during the whole 14-day osteogenic differentiation period as described (Wang et al., 2022). Osteogenic medium with (CHIR99021) and without (DMSO) medium was replenished twice per week, respectively.

# High-throughput image acquisition for β-catenin nuclear translocation and DDAOG senescence staining

BMSCs freshly isolated from young *Wt* and *Prog-Tg* animals were seeded on plastic dishes and after five days of incubation either mock treated (DMSO) or treated with 2,5 µM of CHIR99021 (CHIR) for 24 hours as described (Wang et al., 2022). After PFA fixation, immunofluorescence staining using anti-β-catenin (Santa Cruz, sc-7963) antibody followed by incubation with DNA stain Hoechst was performed. BMSCs were imaged in an automated fashion using 10x objective, with Hoechst (excitation:370/40 nm, emission: 450/60 nm) and TRITC (excitation: 530/45 nm, emission: 594/40 nm) and acquired using ImageXpress Pico imaging system (Molecular Devices). After completion of automated image acquisition, CellReporterXpress® Software (Version 2.5) featuring multi-wavelength cell scoring for nuclear staining and beta-catenin red signals were used for analysis on beta catenin nuclear localization. For the analysis, 4 random ROIs (5mm^2^) each containing approximately 3000 cells were analyzed per well and the average mean intensity per well were presented as a data point. Each biological replicate was analyzed in a technical triplicate.

For DDAOG acquisition, *Wt, Prog-Tg* and doxorubicin-treated lung endothelial cells were incubated with 1 µM bafilomycin for 30 min, followed by one hour incubation either with vehicle only or with a far-red DDAOG probe at 37°C protected from light for staining of senescent cells as described above. ECs were imaged in automated fashion in two channels. Images were taken at 10x magnification and fluorescence color channels: Hoechst (excitation:370/40 nm, emission: 450/60 nm) and cy5 (excitation: 630/40 nm, emission: 695/45 nm). For the analysis, 3-4 random ROIs (5mm^2^) each containing approximately 2000 cells were analyzed per well and the positive cell average area per ROIs were presented as a data point.

The whole well area was analyzed using CellReporterXpress software for assessment of positive cell average area of DDAOG far-red signals.

# Osteoclast differentiation

Bone marrow cells were differentiated to osteoclast lineages according to previously described methods (Cheng et al., 2012). Briefly, bone marrow cells collected from long bones of *Wt* and *Prog-Tg* animals (age 30-35 weeks) were cultured for 24 h, the supernatant containing the hematopoietic stem cells, collected, and seeded at 2.72*10^6^ cells/cm^2^. Cells were cultured in DMEM supplemented with 10% FCS and 50 U/ml penicillin, 50 μg/ml streptomycin, 50 ng/ml TNFSF11 (RANKL; Sigma Aldrich, #R0525) and 25 ng/ml m-CSF (Thermo Fisher Scientific, #RP-8615). After seven days, tartrate-resistant acid phosphatase (TRAP) staining was performed according to manufacturer’s instructions (Sigma-Aldrich, #387A). The number and size of TRAP-positive multinucleated cells (considered as osteoclasts) were measured using thresholding tools in Fiji (Schindelin et al., 2012). All experiments were performed at normoxic conditions (20% oxygen).

# Colony forming unit assay (CFU-F)

Bone marrow mesenchymal stem cells (BMSCs) were isolated from the bone marrow (BM) of long bones of *Wt, Prog-Tg* and *LA-Tg* animals (age ≤14 days) and colony forming unit assay performed as adapted from (Chou et al., 2009). Briefly, BM was flushed using a 21-gauge needle and filtered through a 70 µm cell strainer to obtain a single cell suspension. After centrifugation, cells were seeded at ~4.5*10^5^ cells/cm^2^ in DMEM supplemented with 10% FCS, 50 U/ml penicillin, and 50 μg/ml streptomycin. After 24 h, non-adherent cells were removed and adherent single cells were allowed to form colonies for 10 days with medium changes twice a week. After 10 days formed colonies were visualized after fixing in methanol/acetone (1:1) for 5 minutes, followed by three time washing with PBS and subsequently staining using a 1% crystal violet solution in distilled water for 15 min at RT. After 15 min, colonies were washed three times with distilled water to remove excess staining solution. After acquisition using 10X objective (Olympus BX63 imaging system) the number of colony forming units (CFU) was evaluated in a blinded fashion using Image J (Fiji) with the cell counter plugin.

# RNA isolation and gene expression analysis

Total RNA and total miRNA from tissue and cells was isolated using the miRNeasy Mini Kit (Qiagen, #217084) and quantified using spectrophotometer (NanoDrop Technologies, ND-1000). For mRNAs, cDNA generation was performed using the GoScript™ Reverse Transcription Kit (Promega, #A2791) and for microRNAs, miRCURY LNA^TM^ RT Kit (Qiagen, #339340) followed by qPCR using KAPA SYBR Green PCR master mix (Peqlab, #KK4618) and miRCURY LNA^TM^ SYBR Green Kit (Qiagen, #339346), respectively. Hypoxanthine phosphoribosyltransferase 1 served as reference gene. As reference miRNAs, snoU6, miR 191 and UniSp4 were used. Primers for miRNA qPCR were purchased from Qiagen. All experiments were performed on a StepOnePlus real-time PCR system (Applied Biosystems). Primer sequences are listed in Supplementary table 1.

# Isolation of extracellular vesicles from conditioned media and gene expression analysis

At least 1 ml of EC conditioned media (see above) were used for isolation of total extracellular vesicles with subsequent RNA extraction using exoRNeasy Midi Kit according to manufacturer’s instructions (Qiagen, #77144). Gene expression analysis was performed as described above.

# Bone protein extract preparation and Western Blot / Immunoblotting

For preparation of bone protein extracts, tibiae from *Wt* and *Prog-Tg* animals were dissected, cleaned from soft tissues and separated from the flushed bone marrow. Long bones were homogenized using ceramic beads (Bertin 972 Instruments, P000911-LYSK0-A) in Laemmli sample buffer (Sigma, S3401) supplemented with 4% sodium dodecyl sulfate (SDS), 20% glycerol, 10% 2-mercaptoethanol, 0.004% bromophenol blue and 0,125 M Tris-HCl, pH 6.8. Bone protein extracts were separated using SDS-PAGE and transferred to nitrocellulose membrane for immunoblotting as previously described (Osmanagic-Myers et al., 2019). Subsequently, blocking in 5% bovine serum albumin (BSA) in Tris buffer saline (TBS, 200 mM Tris and 1500 mM sodiumchloride) with 0.05% Tween-20 (TBS-T) was performed followed by overnight incubation with primary antibodies (see below) at 4°C and then 1 h incubation in goat-anti mouse or goat anti-rabbit horseradish peroxidase-coupled secondary antibodies in TBS-T (1:20.000, Jackson Laboratories). Enhanced chemiluminescence (ECL) horseradish peroxidase substrate was used for detection of protein bands (Thermo Fisher, #34076). Following primary antibodies were used: rabbit anti-phospho-serine9-GSK3β−pSer9 (1:300, Invitrogen; #C.367.3) and mouse anti-tubulin ß (1:300, Sigma #T4026) as loading control. Protein bands were detected using ChemiDoc MP Imaging System (Bio-Rad) and quantified using Bio-Rad Image Lab Software.

# µCT analysis and nanoCT

Cortical microstructure as well as bone mineral density were analyzed using µCT (MicroCT 35, SCANCO Medical AG, Switzerland) according to our previously established methods (Papageorgiou et al., 2020). Briefly, µCT analysis was performed at the midshaft of the femur and tibia over a length of 5% proximal and 5% distal from the middle of the bone in *Prog-Tg* and *Wt* littermate animals at the age of 35-40 weeks. The X-ray tube was operated at 70 kV with an intensity of 114 μA, and an exposure time of 800 ms, resulting in a resolution of 10 μm/pixel. Tissue mineral density of the cortex was evaluated using standard Scanco evaluation scripts as we described previously (Papageorgiou et al., 2020). Trabecular microstructure was assessed at the distal femur, proximal tibia and fourth lumbar spine vertebral body (L4). All parameters are reported according to the American Society of Bone and Mineral Research as previously described (Papageorgiou et al., 2020).

Nano-CT measurements were performed according to our established protocols (Weigl et al., 2021). Briefly, nanoCT scans at tibiae of *Prog-Tg* and *Wt* littermate animals (age = 30-35 weeks) using a high-resolution system (nano-CT, μCT 50, SCANCO Medical AG) were performed at 55 kVp, 114 μA, 0.5 mm aluminum filter, 1500 projections/180° with an integration time of 750 ms, averaging 7 for approximately 900 slices (1.8 mm) and reconstructed to an isotropic resolution of 2 μm. On the tibia, scans were centered 2.5 mm proximally of the proximal aspect of the fusion between tibia and fibula. Segmentation was performed using a specifically developed ruleset in Definiens Developer XD 2.7 software (Definiens AG, Munich, Germany). Vessel measurements were performed on the segmented images in Fiji using the BoneJ plugin (Schindelin et al., 2012).

# nanoCT measurements of cortical thickness and vascular volume fraction

Using Fiji (IJ v1.53t) scans were downsampled 50% to an isotropic resolution of 4 µm. Thick vessels (>50 µm) in the cortical part and large trabecular structures present in some scans were manually masked. Scans were thresholded with a threshold of 600 mgHA/cm³. 3D binary close labels (Ollion et al., 2013) was applied with a radius of 8 pixels followed by 3 iterations of dilate and erode to cover small vessels in the cortical part. The binary stack was inverted and 3D binary close labels with a radius of 8 pixels was applied on the inverted stack, closing off thin protrusions attached to the cortex. Using the local thickness tool, each voxel in the cortex was set to the diameter of the largest sphere contained in the cortex containing the voxel. All voxels on the thickness map contained in the segmented cortical part were averaged to measure the cortical thickness (Ct.Th).

Vascular volume fraction measurements were performed on the segmented images in Fiji using the BoneJ plugin. First, scans were downsampled to 50% (4 µm/voxel) and thresholded with a threshold of 500 mgHA/cm³. The largest resulting object in the scan was classified as bone. This classification was copied to the full resolution image. To eliminate errors on the surface due to the difference in resolution, bone was grown 2 times into void where it is ≥ 500 mgHA/cm³. Then void was grown 2 times into bone where it is ≤ 750 mgHA/cm³ resulting in a smooth surface. A difference of Gaussian (DoG) filtration was applied by subtracting a 3D gauss filter with a sigma of 14 µm from a 3D gauss filter with a sigma of 6 µm. Voxels in bone with an intensity of ≤ 500 mgHA/cm³ were classified as vessels while voxels in bone with a DoG ≤ -100 mgHA/cm³ were classified as osteocyte lacunae. A number of correction steps were then performed to eliminate any misclassifications. Osteocytes in contact with vessels were added to the vessel class. Bone voxels with more than 25% pore in a 6*6*6 µm cube centered on the voxels were added to the osteocyte class. Osteocytes with a volume ≥ 3,200 µm³ were reclassified as vessels. Osteocytes with both a length of ≥ 30 µm and a length/width ratio of ≥ 4 were reclassified as vessels. To eliminate classification errors on the surface of the bone, osteocytes and vessels in contact with void were removed if they extended ≤ 8 µm into the bone by coating the void 8 µm into osteocyte and vessel classes with a temporary class and then growing back osteocyte and vessel classes into the temporary class. Any remaining temporary class was then reclassified as void. Bone volume, vessel number and vessel area were measured. A classification image was exported in png format with each class with a different color.

The classification image was imported into Fiji. The color classification images converted into a binary image which included only the vessels. In the vessel image, the BoneJ thickness tool was used to measure vessel thickness. The vessels were skeletonized and vessel length was calculated from the skeleton. The vast majority of vessels observed had a diameter of ≤ 20 µm. However, in some samples, single vessels with a diameter of up to 85 µm were observed which would dramatically shift the measurements of vessel volume and thickness where such vessels are present. For this reason, a second measurement of volume and thickness was performed which excluded vessels with a diameter ≥ 40 µm.

# Bone immunohistochemistry and immunofluorescence

Freshly dissected tibiae from young (age ≤14 days) and adult animals (age~30-35 weeks) were fixed in ice-cold 4% paraformaldehyde (PFA) overnight, decalcified manually in 0.5 M EDTA solution for 1 week at 4°C, paraffin embedded and 2 µm section were cut using microtome (Micros Austria). For preparation of cryosections, dissected tibiae were fixed for 24 h in 4% paraformaldehyde (PFA) followed by decalcification in 10% EDTA decalcifying solution (Milestone, #EUH210) at 37°C for 7 h with constant magnetic rotation using KOS, the Multifunctional Microwave Tissue Processor (Milestone). Decalcified bone tissues were equilibrated in 30% sucrose overnight for cryoprotection followed by embedding in Cryo Glue medium (Slee medical GmbH, #30001101), snap-freezing using 2-Methylbutane (Roth, #3927.2) in liquid nitrogen and immediate storage at -80°C. Cryosections were generated using Cryostat Microm HM 500 OM.

Hematoxylin and eosin (HE) staining was performed as previously described (Schmidt et al., 2012). TRAP staining of tibia sections was performed according to manufacturer´s protocol (Sigma-Aldrich, 387A). For immunofluorescence staining, antigen retrieval was performed in TE buffer (10 mM Tris, 1 mM EDTA) overnight at 60°C and subsequent staining with primary antibodies at 4°C overnight using mouse-on-mouse (MOM) basic detection Kit (Vector Laboratories, #BMK-2202) using biotinylated anti-mouse IgG antibodies and fluorophore conjugated streptavidin according to manufacturer’s instructions. Following primary antibodies were used: mouse monoclonal anti-human Lamin A/C (JOL2) (1:50; clone JoL2, mab3211, abcam), rabbit polyclonal anti-mouse Sp7 (1:100; Abcam, ab22552), goat polyclonal anti-mouse Lepr (1:30; R&D systems, AF497), rat anti-mouse endomucin-PE or endomucin unconjugated (1:50; SantaCruz Biotechnology, sc-65495). After washing with PBS, tissue sections were incubated with fluorophore-labeled secondary antibodies or streptavidin for one hour at room temperature. Following secondary antibodies were used: goat anti-rabbit Alexa 488 (Lepr, Invitrogen), donkey anti-rabbit Cy5 (Sp7; Jackson labs), donkey anti-rat Alexa 594 (uncoupled Emcn, Invitrogen) or streptavidin Alexa Fluor 488 (Jol, Jackson labs). In case of single Jol staining, streptavidin 594 (Jackson labs) was used. For staining of DNA, specimens were incubated with Hoechst (1:5000 in 0.1% Tween-PBS) for 10 min at room temperature. Sections were mounted in mowiol. Images were acquired using Olympus BX61VS Upright Fluorescence Microscope, 40x objective and VSI software. Images were analyzed using Image J (Fiji).

# Image acquisition and quantitative analysis of osteocyte lacunae, adipocytes, TRAP, vascularization, SP7+osteoprogenitors and Lef1-staining

The osteocyte lacunae were assessed on HE-stained tibiae sections with those clearly lacking well-defined nuclei characterized as empty osteocyte lacunae as previously described (Hemmatian et al., 2017; Schmidt et al., 2012). Empty osteocyte lacunae were presented in percentage of total number of counted lacunae. Six regions of interest (ROIs) per animal were analyzed in identical regions of the diaphysis from young and adult mice by 2 observers in a blinded manner. Similarly, on HE-stained tibiae sections adipocytes, identified as white regular formed blobs, were manually counted which was normalized to the total assessed area according to previous published methods (Liu et al., 2011).

Analysis of TRAP staining was carried out in the diaphysis of adult animals in 6 different ROIs per animal and in the trabecular region in 3 ROIs with each ROI covering an area of 1.24 mm^2^. Subsequently the number of TRAP positive cells was normalized to the total area of the analyzed region.

For quantification of blood vessels, endomucin-stained positive areas, determined using thresholding method in Image J (Fiji), were normalized to total area. The vasculature was analyzed in the metaphysis, endosteal niche lining the cortical bone and in the bone marrow. For each area, 4-5 ROIs (0.31 mm^2^/ROI) were analyzed per slide and the average of positive-stained endomucin area of all ROIs per slide is presented as single data point. Three consecutive sections were analyzed per animals in three different animals.

Quantification of Sp7 (osterix) positive cells was performed manually in 2-3 ROIs per slide of the metaphysis and 2-3 ROIs per slide in the cortical bone of the diaphysis. The number of SP7^+^ cells in the metaphysis and in cortical bone were analyzed in an area covering 0.31 mm^2^ per ROI. Three serial slides were analyzed per animals in three different animals.

Quantification of Lepr positive cells was performed in 3-4 ROIs per animal in an area of 1.24 mm^2^ of the bone marrow within the diaphysis of adult animals and normalized to the area that was assessed.

For quantification of Lef1, a total number of six ROIs from two independent BMSC isolations were analyzed using 20x objective (Olympus BX63). Intensity of Lef1-signals were measured either across whole cell area (total Lef1) or Hoechst-stained nuclear area (nuclear Lef1) using Image J (Fiji). Image analysis was performed with Fiji software (NIH) in a blinded manner (Schindelin et al., 2012).

# ELISA

Quantification of the procollagen type 1 N-terminal propeptide (PINP1) in mouse EDTA plasma samples was performed using ELISA kit according to manufacturer’s instructions (Biovendor Immunodiagnostics, AC-33F).

# Apoptosis assays

For assessment of apoptosis in differentiated osteoblast populations, real-time monitoring of apoptosis was performed for 48 h during *in vitro* BMSC osteogenic differentiation using RealTime-Glo™ Annexin V Apoptosis and Necrosis Assay according to manufacturer’s instructions (Promega, #JA1011). Briefly, ~2.2*10^6^ cells/cm^2^ enriched BMSC populations derived from young *Wt* and *Prog-Tg* mice (≤ day 14) were seeded in a 96-well plate format and subjected to osteogenic differentiation. After five days, detection reagent containing annexinV-complementary subunits of NanoBIT luciferase with time-released luciferase substrate and profluorescent DNA dye were added. Apoptosis was detected by measuring luminescence intensity after 48 h incubation after 7 days of differentiation. The extent of apoptosis is proportional to annexin V protein binding to the phosphatidylserine (PS) on the outer leaflet cell membranes. Necrosis was quantitated by measuring fluorescence of the DNA binding dye upon loss of cell membrane integrity. For positive controls, cells were treated for 48 h either with 0.5 µM Saturosporine (Cell Signaling, #9953) to induce apoptosis or with 1 µM Ionomycin (Sigma, #I3909) to induce necrosis in the presence of the detection reagent. Luminescence and fluorescence signals were recorded using BioTek Microplate Reader and results were expressed as relative luminescence units (RLU) and relative fluorescence units (RFU) normalized to the blank values.

For assessment of apoptosis on whole tibiae sections Terminal deoxynucleotidyl Transferase (TdT) dUTP nick end labelling (TUNEL) staining procedure was performed according to manufacturer’s instructions (TUNEL Assay Kit - HRP-DAB; Abcam #Ab206386). Briefly, re-hydrated paraffin embedded tibiae sections from young (age ≤14 day) and adult (age~30-35 weeks) mice were incubated with TdT labelling mix containing TdT enzyme and biotin-labelled deoxynucleotides. Subsequently, strepatavidin-bound horseradish peroxidase conjugate was added followed by diaminobenzidine (DAB) reaction to detect DNA fragmentation as brown substrate. For positive controls, slides were treated with DNAse (300 U/ml; Qiagen, #79254) for 30 min at RT.

**miRNA sequencing from HGPS plasma samples and bioinformatics analysis**

Plasma HGPS patient samples were obtained from The Progeria Research Foundation (PRF) Cell and Tissue Bank. The HGPS plasma samples were 009 (m, 8.5 y), 204 (f, 5 y), 194 P1 (m, 10.2 y), 230 (f, 8.6 y); the unaffected age-and gender matched control plasma samples were 612p (m, 8.8 y), 555p (f, 4.8 y), 648p (m, 10.1 y), 624p (f, 8.6 y). RNA extraction from plasma samples, small RNA library preparation and data analysis were performed by TamiRNA according to established methods (Weigl et al., 2021). Briefly, small RNA library preparation was performed using CleanTag™ Library Preparation Kit (TriLink). Library quality and quantity was controlled by capillary electrophoresis (Agilent). Following equimolar pooling, libraries were purified using preparative capillary electrophoresis with enrichment of RNA insert sizes (18 - 36bp), which corresponds to mature microRNAs. miR-sequencing was performed on the Illumina HiSeq2000 at Vienna Biocenter Core Facilities (VBCF). The short sequencing reads were aligned against the Homo sapiens (hsa, TXID: 9606). Roughly ~25 million short reads per replicate were generated with a microRNA mapping score of approximately ~10%. Data analysis was performed using the miND pipeline.

# Bioinformatics analysis for miRNA sequencing from HGPS plasma samples

Reads from all passing samples were adapter trimmed and quality filtered using cutadapt v2.3 and filtered for a minimum length of 17nt. Reads were mapped first against the genomic reference GRCh38.p12 provided by Ensembl allowing for two mismatches, and subsequently to miRBase v22.1 that was filtered for miRNAs of human origin only, allowing for one mismatch. Statistical analysis of preprocessed NGS data was done with R v3.6 and the packages pheatmap v1.0.12, pcaMethods v1.78 and genefilter v1.68. Differential expression analysis with edgeR v3.28 used the quasi-likelihood negative binomial generalized log-linear model functions provided by the package. The independent filtering method of DESeq2 was adapted for use with edgeR to remove low abundant miRs and thus optimize the false discovery rate (FDR) correction (Diendorfer et al., 2022).

# Statistics and reproducibility

All experiments were repeated as indicated with n depicting biological replicates except if others stated in the figure legends. Data are presented as mean ± SEM or median with minimum and maximum values. Data analysis was performed in a blinded manner. Sample sizes for mouse numbers were predetermined according to our previous estimates. For ~10% effect size shown in µCT measurements using a=0.05 and 80% power ~7-10 animals are needed. Statistical analyses were performed using GraphPad Prism version 7.05 for Windows, GraphPad software, La Jolla California USA. Comparison of two groups was done by unpaired Student´s two-tailed t-test. For antimiR and scramble treated samples paired Student´s two-tailed t-test was used. For non-normally distributed data, Mann Whitney rank sum test was used. Analysis of more than two groups was done using one-way ANOVA followed by posthoc Tukey´s multiple comparisons test or in case of non-normally distributed data Kruskal-Wallis test with posthoc Dunn´s multiple comparison test was used. Data were considered significantly different if p<0.05.

# Study approval

All mouse experiments were approved (No: 2020-0.469.732; July 2020) by the regional Ethics Committee for Laboratory Animal Experiments at the Medical University of Vienna and the Austrian Ministry of Science Research and Economy (66.009/0321-WF/V/3b/2016, according to Austrian Law BGBI. I Nr.114/2012 (TVG2012) and in accordance with the Guide for the Care and Use of Laboratory Animals published by the US National Institutes of Health (NIH Publication No. 85-23, revised 1996). S.OM. has from the side of Progeria Research Foundation (PRF) an approved “Material Transfer Agreement” and Application and Agreement for Cells, DNA or Tissue HGPS and control plasma samples (11/12/2019). PRF harbors ethical approval for human research from Rhode Island Hospital's Institutional Review Board, for which all donors have given consent. PRF fulfills all standards to protect donors and their material.

**Supplementary Table 1. Trabecular bone parameters assessed by µCT measurements in tibia and lumbar vertebral body 4 (L4).**

| **Tibia** | | | | | | | |
| --- | --- | --- | --- | --- | --- | --- | --- |
|  | *Wt* | *Prog-Tg* | p value  *Wt* vs. *Prog-Tg* | *LM_LA-Tg* | *LA-Tg* | p value  *LM_LA-Tg* vs. *LA-Tg* | p value  *Prog-Tg* vs. *LA-Tg* |
| TV  [mm3] | 1,34 ± 0,47 | 1,40 ± 0,35 | ns | 1,75 ± 0,23 | 1,58 ± 0,50 | ns | ns |
| BV  [mm3] | 0,09 ± 0,04 | 0,10 ± 0,04 | ns | 0,19 ± 0,07 | 0,26 ± 0,14 | ns | ns |
| BV/TV | 0,08 ± 0,03 | 0,07 ± 0,02 | ns | 0,11 ± 0,03 | 0,15 ± 0,07 | ns | ns |
| Tb.N  [1/mm] | 1,93 ± 0,01 | 1,96 ± 0,77 | ns | 2,51 ± 0,60 | 3,35 ± 1,36 | ns | ns |
| Tb.Sp  [mm] | 0,04 ± 0,01 | 0,04 ± 0,01 | ns | 0,04 ± 0,01 | 0,05 ± 0,01 | ns | ns |
| Tb.Th  [mm] | 0,61 ± 0,30 | 0,59 ± 0,33 | ns | 0,40 ± 0,11 | 0,31 ± 0,15 | ns | ns |
| BMD  [mgHA/ccm] | 844 ± 55 | 847 ± 27 | ns | 852 ± 10 | 850 ± 11 | ns | ns |
| **L4 (4^th^ vertebral body)** | | | | | | | |
|  | *Wt* | *Prog-Tg* | p value  *Wt* vs. *Prog-Tg* | *LM_LA-Tg* | *LA-Tg* | p value  *LM_LA-Tg* vs. *LA-Tg* | p value  *Prog-Tg* vs. *LA-Tg* |
| TV  [mm3] | 2,79 ± 0,28 | 2,64 ± 0,33 | ns | 2,85 ± 0,24 | 2,71 ± 0,28 | ns | ns |
| BV  [mm3] | 0,44 ± 0,10 | 0,41 ± 0,11 | ns | 0,44 ± 0,05 | 0,62 ± 0,22 | ns | ns |
| BV/TV | 0,16 ± 0,04 | 0,16 ± 0,04 | ns | 0,16 ± 0,01 | 0,22 ± 0,06 | ns | ns |
| Tb.N  [1/mm] | 4,24 ± 0,96 | 4,26 ± 1,1 | ns | 3,89 ± 0,20 | 5,09 ± 0,96 | ns | ns |
| Tb.Sp  [mm] | 0,04 ± 0,01 | 0,04 ± 0,01 | ns | 0,04 ± 0,01 | 0,04 ± 0,01 | ns | ns |
| Tb.Th  [mm] | 0,21 ± 0,06 | 0,21 ± 0,06 | ns | 0,22 ± 0,01 | 0,16 ± 0,05 | ns | ns |
| BMD  [mgHA/ccm] | 861 ± 34 | 897 ± 35 | ns | 879 ± 8 | 898 ± 11 | ns | ns |

*Prog-Tg*, *LA-Tg* and corresponding *Wt* littermates (*LM*) were analyzed (age=35-40 weeks; n=8 *Prog-Tg,* n=9 *Wt* littermates (*Wt*), n=5 *LA‑Tg,* and n=5 for corresponding *Wt* littermate (*LM_LA-Tg*)). TV, total volume; BV, bone volume; BV/TV, bone volume fraction; Tb.N, trabecular number; Tb.Sp, trabecular spacing; Tb.Th, trabecular thickness; BMD, bone mineral density. Data presented as mean ± SD. Statistical analysis by one-way ANOVA followed by multiple comparisons post-hoc Tukey test (ns. not significant).

**Supplementary Table 2 (List of DE miRs in HGPS)**

| Human HGPS patient plasma | | | | |
| --- | --- | --- | --- | --- |
|  | **miRNA** | **logFC** | **P.Value** | **adj.P.Val** |
| 1 | hsa-miR-155-5p | 2,666655236 | 1.52857576656019e-9 | 3.06115593211956e-7 |
| 2 | hsa-miR-150-5p | 3,180185332 | 2.45875978483499e-9 | 3.06115593211956e-7 |
| 3 | hsa-miR-146b-5p | 1,774842505 | 4.69161887205674e-7 | 3,89404E-05 |
| 4 | hsa-let-7b-5p | -1,095095223 | 6,64395E-06 | 0,000413586 |
| 5 | hsa-miR-451a | -1,626976442 | 1,90898E-05 | 0,000950671 |
| 6 | hsa-miR-1275 | 4,691456033 | 7,15129E-05 | 0,002967785 |
| 7 | hsa-let-7c-5p | -1,766830743 | 0,000114145 | 0,004060315 |
| 8 | hsa-miR-98-5p | 1,705115068 | 0,000213844 | 0,006655908 |
| 9 | hsa-miR-574-3p | 11,4868459 | 0,00101718 | 0,025421394 |
| 10 | hsa-miR-181a-5p | 1,101781106 | 0,001113789 | 0,025421394 |
| 11 | hsa-miR-143-3p | -1,656073479 | 0,001123033 | 0,025421394 |
| 12 | hsa-miR-107 | -1,177396571 | 0,001621058 | 0,032877987 |
| 13 | hsa-miR-16-5p | -1,214497177 | 0,001805155 | 0,032877987 |
| 14 | hsa-miR-223-3p | 2,234108107 | 0,001848562 | 0,032877987 |
| 15 | hsa-miR-28-3p | 1,302297078 | 0,002279135 | 0,037833635 |
| 16 | hsa-miR-181b-5p | 1,449041649 | 0,002869786 | 0,044661052 |
| 17 | hsa-miR-361-3p | 3,101923584 | 0,003127523 | 0,04580902 |
| 18 | hsa-miR-30a-5p | -1,299498844 | 0,003464378 | 0,047923893 |
| 19 | hsa-miR-342-5p | 2,216299809 | 0,003765935 | 0,049211917 |
| 20 | hsa-miR-28-5p | 2,919435772 | 0,003952764 | 0,049211917 |
| 21 | hsa-miR-342-3p | 2,465246186 | 0,007487054 | 0,088775064 |
| 22 | hsa-miR-144-3p | -2,009842363 | 0,008110307 | 0,091793934 |
| 23 | hsa-miR-363-3p | -1,488493233 | 0,011419734 | 0,123631039 |
| 24 | hsa-miR-181c-3p | 10,64230672 | 0,013232144 | 0,135922549 |
| 25 | hsa-miR-92b-3p | -1,246767309 | 0,014099382 | 0,135922549 |
| 26 | hsa-miR-320b | -1,268988196 | 0,014192716 | 0,135922549 |
| 27 | hsa-miR-182-5p | -1,335670097 | 0,016617954 | 0,153254464 |
| 28 | hsa-miR-423-5p | -0,847418855 | 0,0174014 | 0,154748163 |
| 29 | hsa-miR-487b-3p | 10,44717206 | 0,019518934 | 0,167593606 |
| 30 | hsa-miR-103a-3p | -0,779991836 | 0,020198265 | 0,167645601 |
| 31 | hsa-miR-4286 | 10,38658439 | 0,021748578 | 0,174690193 |
| 32 | hsa-miR-93-5p | -0,919140396 | 0,026342898 | 0,202694247 |
| 33 | hsa-miR-144-5p | -1,380680231 | 0,026863093 | 0,202694247 |
| 34 | hsa-miR-486-3p | -1,294309472 | 0,030698921 | 0,21379621 |
| 35 | hsa-miR-183-5p | -1,354197797 | 0,032057748 | 0,21379621 |
| 36 | hsa-miR-423-3p | 1,038097391 | 0,032063084 | 0,21379621 |
| 37 | hsa-miR-7-1-3p | 10,13345088 | 0,032970196 | 0,21379621 |
| 38 | hsa-miR-4508 | -1,409521039 | 0,033358405 | 0,21379621 |
| 39 | hsa-miR-185-5p | -1,800163242 | 0,033486153 | 0,21379621 |
| 40 | hsa-miR-146a-5p | 0,832477611 | 0,036356885 | 0,219978381 |
| 41 | hsa-miR-889-3p | 10,05188404 | 0,036909233 | 0,219978381 |
| 42 | hsa-miR-21-3p | 10,03874157 | 0,037104787 | 0,219978381 |
|  | **miRNA** | **logFC** | **P.Value** | **adj.P.Val** |
| 43 | hsa-miR-101-3p | -0,944633022 | 0,040089756 | 0,232147659 |
| 44 | hsa-miR-31-5p | 9,895529525 | 0,044573152 | 0,252243517 |
| 45 | hsa-miR-766-3p | 6,379366437 | 0,045592407 | 0,252277984 |
| 46 | hsa-miR-181a-2-3p | 1,651165437 | 0,049521703 | 0,263252981 |
| 47 | hsa-miR-382-3p | 3,916862637 | 0,049690322 | 0,263252981 |

**Supplementary Table 3. Primers used for quantitative real-time PCR analysis.**

| Gene | Genbank accession number | Primer Sequences |
| --- | --- | --- |
| *Hprt* | NM_013556.2 | Forward: 5’-GCAGTCCCAGCGTCGTGATTA-3’  Reverse: 5’-TGATGGCCTCCCATCTCCTTCA-3’ |
| *Runx2* | NM_001146038.2 | Forward: 5’-CGAAATGCCTCCGCTGTTAT-3’  Reverse: 5’-TGTCTGTGCCTTCTTGGTTCC-3’ |
| *Sp7* | NM_130458.4 | Forward: 5’-GTCCTCTCTGCTTGAGGAAGAA-3’  Reverse: 5’-GGGCTGAAAGGTCAGCGTAT-3’ |
| *Col1a1* | NM_007742.4 | Forward: 5’-GGTCCACAAGGTTTCCAAGG-3’  Reverse: 5’-GTTCCAGGCAATCCACGAG-3’ |
| *Alpl* | NM_007431.3 | Forward: 5’-CCTGACTGACCCTTCGCTCT-3’  Reverse: 5’-CCATCTCCACTGCTTCATGC-3’ |
| *Ocn* | NM_007541.3 | Forward: 5’-GCAGGAGGGCAATAAGGTAG-3’  Reverse: 5’-CTTTAGGGCAGCACAGGTC-3’ |
| *Dmp1* | NM_001359013.1 | Forward: 5’-GTTCCTTTGGGGGCTGTC-3’  Reverse: 5’-CTATTTGCCTGTCCCTCTGG-3’ |
| *Mepe* | NM_053172.2 | Forward: 5’- TGTTGGACTGCTCCTCTTCA-3’  Reverse: 5’- CCATCCTCTGTGCCTTCATC-3’ |
| *Sost* | NM_024449.6 | Forward: 5’-TTCAGGAATGATGCCACAGA-3’  Reverse: 5’-GTCAGGAAGCGGGTGTAGTG-3’ |
| *p16^Ink4a^* | NM_009877.2 | Forward: 5’-AGAGCGGGGACATCAAGAC-3’  Reverse: 5’-CTGAGGCCGGATTTAGCTC-3’ |
| *p21^Cip1^* | NM_007669.5 | Forward: 5’-TGCCAGCAGAATAAAAGGTG-3’  Reverse: 5’-TTGCTCCTGTGCGGAAC-3’ |
| *Trp53* | NM_011640.3 | Forward: 5’-ACAGCACATGACGGAGGTC-3’  Reverse: 5’-CTCGGGTGGCTCATAAGGTA-3’ |
| *Il1a* | NM_010554.4 | Forward: 5’-TGCCATTGACCATCTCTCTCT-3’  Reverse: 5’-GATACTGTCACCCGGCTCTC-3’ |
| *Il6* | NM_031168.2 | Forward: 5’-GGGAAATCGTGGAAATGAGA-3’  Reverse: 5’-TCCAGTTTGGTAGCATCCATC-3’ |
| *Tnfa* | NM_013693.3 | Forward: 5’-CCCCAAAGGGATGAGAAGTT-3’  Reverse: 5’-TGGGCTACAGGCTTGTCACT-3’ |
| *Lef1* | NM_010703.5 | Forward: 5’-AGCCTGTTTATCCCATCACG-3’  Reverse: 5’-GGGTGCTCCTGTTTGACCT-3’ |
| *Fzd3* | NM_021458.2 | Forward: 5’-GAAGCAAAGCAGGGAGTGTC-3’  Reverse: 5’-CTCCATTCCTCGGTAACTGC-3’ |
| *Ctsk* | NM_007802.4 | Forward: 5’-TAGCCACGCTTCCTATCCGA-3’  Reverse: 5’-CCGAGAGATTTCATCCACCTTG-3’ |
| *Nfatc1* | NM_016791.4 | Forward: 5’-CCCGGAGTTCGACTTCGATT-3’  Reverse: 5’-CATAACTGTAGTGTTCTGCGGC-3’ |
| *Tnfrsf11b* | NM_008764.4 | Forward: 5’-AGCTGCTGAAGCTGTGGAAA-3’  Reverse: 5’-CTGCTCTGTGGTGAGGTTCG-3’ |
| *Tnfsf11* | NM_011613.4 | Forward: 5’-TTGCACACCTCACCATCAAT-3’  Reverse: 5’-CCCTTAGTTTTCCGTTGCTT-3’ |
| *Il1b* | NM_008361.4 | Forward: 5’-GCCACCTTTTGACAGTGATGAG-3’  Reverse: 5’-TGATGTGCTGCTGCGAGATT-3’ |
| *Ccl20* | NM_016960.2 | Forward: 5’-TCCTTGCTTTGGCATGGGTA-3’  Reverse: 5’-TCTTAGGCTGAGGAGGTTCACA-3’ |

**References**

Chen, C., Sun, M. A., Warzecha, C., Bachu, M., Dey, A., Wu, T., Adams, P. D., Macfarlan, T., Love, P., & Ozato, K. (2020). HIRA, a DiGeorge Syndrome Candidate Gene, Confers Proper Chromatin Accessibility on HSCs and Supports All Stages of Hematopoiesis. *Cell Rep*, *30*(7), 2136-2149 e2134. <https://doi.org/10.1016/j.celrep.2020.01.062>

Cheng, S., Zhao, S. L., Nelson, B., Kesavan, C., Qin, X., Wergedal, J., Mohan, S., & Xing, W. (2012). Targeted disruption of ephrin B1 in cells of myeloid lineage increases osteoclast differentiation and bone resorption in mice. *PLoS One*, *7*(3), e32887. <https://doi.org/10.1371/journal.pone.0032887>

Chou, M. Y., Yan, D., Jafarov, T., & Everett, E. T. (2009). Modulation of murine bone marrow-derived CFU-F and CFU-OB by in vivo bisphosphonate and fluoride treatments. *Orthod Craniofac Res*, *12*(2), 141-147. <https://doi.org/10.1111/j.1601-6343.2009.01447.x>

Debacq-Chainiaux, F., Erusalimsky, J. D., Campisi, J., & Toussaint, O. (2009). Protocols to detect senescence-associated beta-galactosidase (SA-betagal) activity, a biomarker of senescent cells in culture and in vivo. *Nat Protoc*, *4*(12), 1798-1806. <https://doi.org/10.1038/nprot.2009.191>

Diendorfer, A., Khamina, K., Pultar, M., & Hackl, M. (2022). miND (miRNA NGS Discovery pipeline): a small RNA-seq analysis pipeline and report generator for microRNA biomarker discovery studies [version 1; peer review: 2 approved with reservations]. *F1000 Research*. <https://doi.org/https://doi.org/10.12688/f1000research.94159.1>

Flor, A., Pagacz, J., Thompson, D., & Kron, S. (2022). Far-red Fluorescent Senescence-associated beta-Galactosidase Probe for Identification and Enrichment of Senescent Tumor Cells by Flow Cytometry. *J Vis Exp*(187). <https://doi.org/10.3791/64176>

Gory, S., Vernet, M., Laurent, M., Dejana, E., Dalmon, J., & Huber, P. (1999). The vascular endothelial-cadherin promoter directs endothelial-specific expression in transgenic mice. *Blood*, *93*(1), 184-192. <https://www.ncbi.nlm.nih.gov/pubmed/9864160>

Hemmatian, H., Bakker, A. D., Klein-Nulend, J., & van Lenthe, G. H. (2017). Aging, Osteocytes, and Mechanotransduction. *Curr Osteoporos Rep*, *15*(5), 401-411. <https://doi.org/10.1007/s11914-017-0402-z>

Houlihan, D. D., Mabuchi, Y., Morikawa, S., Niibe, K., Araki, D., Suzuki, S., Okano, H., & Matsuzaki, Y. (2012). Isolation of mouse mesenchymal stem cells on the basis of expression of Sca-1 and PDGFR-alpha. *Nat Protoc*, *7*(12), 2103-2111. <https://doi.org/10.1038/nprot.2012.125>

Kusumbe, A. P., Ramasamy, S. K., & Adams, R. H. (2014). Coupling of angiogenesis and osteogenesis by a specific vessel subtype in bone. *Nature*, *507*, 323-328. <https://doi.org/10.1038/nature13145>

Liu, L. F., Shen, W. J., Ueno, M., Patel, S., & Kraemer, F. B. (2011). Characterization of age-related gene expression profiling in bone marrow and epididymal adipocytes. *BMC Genomics*, *12*, 212. <https://doi.org/10.1186/1471-2164-12-212>

Manakanatas, C., Ghadge, S. K., Agic, A., Sarigol, F., Fichtinger, P., Fischer, I., Foisner, R., & Osmanagic-Myers, S. (2022). Endothelial and systemic upregulation of miR-34a-5p fine-tunes senescence in progeria. *Aging (Albany NY)*, *14*(undefined). <https://doi.org/10.18632/aging.203820>

Ollion, J., Cochennec, J., Loll, F., Escude, C., & Boudier, T. (2013). TANGO: a generic tool for high-throughput 3D image analysis for studying nuclear organization. *Bioinformatics*, *29*(14), 1840-1841. <https://doi.org/10.1093/bioinformatics/btt276>

Osmanagic-Myers, S., Kiss, A., Manakanatas, C., Hamza, O., Sedlmayer, F., Szabo, P. L., Fischer, I., Fichtinger, P., Podesser, B. K., Eriksson, M., & Foisner, R. (2019). Endothelial progerin expression causes cardiovascular pathology through an impaired mechanoresponse. *J Clin Invest*, *129*(2), 531-545. <https://doi.org/10.1172/JCI121297>

Papageorgiou, M., Föger-Samwald, U., Wahl, K., Kerschan-Schindl, K., & Pietschmann, P. (2020). Age- and Strain-Related Differences in Bone Microstructure and Body Composition During Development in Inbred Male Mouse Strains. *Calcified tissue international*, *106*, 431-443. <https://doi.org/10.1007/S00223-019-00652-8>

Poulos, M. G., Ramalingam, P., Gutkin, M. C., Llanos, P., Gilleran, K., Rabbany, S. Y., & Butler, J. M. (2017). Endothelial transplantation rejuvenates aged hematopoietic stem cell function. *The Journal of clinical investigation*, *127*, 4163-4178. <https://doi.org/10.1172/JCI93940>

Rossi, L., Challen, G. A., Sirin, O., Lin, K. K., & Goodell, M. A. (2011). Hematopoietic stem cell characterization and isolation. *Methods Mol Biol*, *750*, 47-59. <https://doi.org/10.1007/978-1-61779-145-1_3>

Sagelius, H., Rosengardten, Y., Hanif, M., Erdos, M. R., Rozell, B., Collins, F. S., & Eriksson, M. (2008). Targeted transgenic expression of the mutation causing Hutchinson-Gilford progeria syndrome leads to proliferative and degenerative epidermal disease. *J Cell Sci*, *121*(Pt 7), 969-978. <https://doi.org/10.1242/jcs.022913>

Schindelin, J., Arganda-Carreras, I., Frise, E., Kaynig, V., Longair, M., Pietzsch, T., Preibisch, S., Rueden, C., Saalfeld, S., Schmid, B., Tinevez, J. Y., White, D. J., Hartenstein, V., Eliceiri, K., Tomancak, P., & Cardona, A. (2012). Fiji: an open-source platform for biological-image analysis. *Nature methods*, *9*, 676-682. <https://doi.org/10.1038/NMETH.2019>

Schmidt, E., Nilsson, O., Koskela, A., Tuukkanen, J., Ohlsson, C., Rozell, B., & Eriksson, M. (2012). Expression of the Hutchinson-Gilford progeria mutation during osteoblast development results in loss of osteocytes, irregular mineralization, and poor biomechanical properties. *J Biol Chem*, *287*(40), 33512-33522. <https://doi.org/10.1074/jbc.M112.366450>

Smith, A. O., Adzraku, S. Y., Ju, W., Qiao, J., Xu, K., & Zeng, L. (2021). A novel strategy for isolation of mice bone marrow endothelial cells (BMECs). *Stem Cell Res Ther*, *12*(1), 267. <https://doi.org/10.1186/s13287-021-02352-3>

Sun, J. F., Phung, T., Shiojima, I., Felske, T., Upalakalin, J. N., Feng, D., Kornaga, T., Dor, T., Dvorak, A. M., Walsh, K., & Benjamin, L. E. (2005). Microvascular patterning is controlled by fine-tuning the Akt signal. *Proc Natl Acad Sci U S A*, *102*(1), 128-133. <https://doi.org/10.1073/pnas.0403198102>

Wang, B., Khan, S., Wang, P., Wang, X., Liu, Y., Chen, J., & Tu, X. (2022). A Highly Selective GSK-3beta Inhibitor CHIR99021 Promotes Osteogenesis by Activating Canonical and Autophagy-Mediated Wnt Signaling. *Front Endocrinol (Lausanne)*, *13*, 926622. <https://doi.org/10.3389/fendo.2022.926622>

Weigl, M., Kocijan, R., Ferguson, J., Leinfellner, G., Heimel, P., Feichtinger, X., Pietschmann, P., Grillari, J., Zwerina, J., Redl, H., & Hackl, M. (2021). Longitudinal Changes of Circulating miRNAs During Bisphosphonate and Teriparatide Treatment in an Animal Model of Postmenopausal Osteoporosis. *J Bone Miner Res*, *36*(6), 1131-1144. <https://doi.org/10.1002/jbmr.4276>
